# The bacterial spectrum of spinal infections based on blood culture, tissue culture, and molecular methods: a systematic review and meta-analysis

**DOI:** 10.1038/s41598-025-28576-4

**Published:** 2025-11-28

**Authors:** Rui Zhang, Mengdie Wang, Xinyu Liu, Feifei Yang, Xinyuan Xu, Liye Zi, Zhi Liang, Xiaorong Liu, Hongbing Gao, Xuesong Chen, Guozhong Zhou

**Affiliations:** 1 Faculty of Life Science and Technology & The affiliated Anning First People’s Hospital, Kunming University of Science and Technology, Kunming, 650302 China; 2 Department of Pain Medicine, The Affiliated Anning First People’s Hospital of Kunming University of Science and Technology, Kunming, 650302 Yunnan China; 3 School of Basic Medical Sciences, Kunming University of Science and Technology, Kunming, 650500 Yunnan China

**Keywords:** Spinal infections, Bacterial infection spectrum, Blood culture, Tissue culture, Molecular biology method, Meta-analysis, Clinical microbiology, Pathogens

## Abstract

Spinal infections (SI) are on the rise due to an aging population and the prevalence of more invasive procedures. This study aims to systematically review the microbiological spectrum of SI to enhance diagnostic accuracy and inform effective antibiotic treatment strategies. The last search was conducted on May 9th, 2024, from databases including EMBASE, PubMed, and Web of Science. The outcome variable is infection rate, and the detection method used should be blood culture, tissue culture, or molecular biology method. Two researchers independently extracted research data and evaluated its quality using the JBI Critical Appraisal Tools. Out of 14,639 identified records, 156 studies (encompassing 13,539 patients) were included. Staphylococcus aureus was identified as the most prevalent pathogen, with pooled infection rates of 17.6% (95%CI: 12.8–22.9%; *I*^*2*^=93%) in blood culture, 16.8% (95%CI: 14.0–19.8%; *I*^*2*^=96%) in tissue culture, and 12.0% (95%CI: 9.3–15.0%; *I*^*2*^=35%) in molecular methods. The bacterial spectrum also featured *Staphylococcus epidermidis*, *Escherichia coli*, and *Mycobacterium tuberculosis* (MTB). Molecular methods, particularly metagenomic next-generation sequencing (mNGS), demonstrated markedly superior sensitivity for MTB detection, with a pooled rate of 9.7% (95%CI: 4.6–16.3%; *I*^*2*^=90%) compared to 1.3% (95%CI: 0.6–2.1%; *I*^*2*^=86%) by tissue culture. The odds ratio for MTB detection with mNGS versus conventional culture was 4.24 (95%CI: 1.68–10.73). This review confirms that a core group of pathogens, including *Staphylococcus aureus, Staphylococcus epidermidis*, MTB, and *Escherichia coli*. Our findings underscore that tissue culture is fundamental for common pyogenic bacteria, while metagenomic next-generation sequencing is indispensable for detecting fastidious organisms like MTB. *Trial registration*: The protocol was registered with PROSPERO (No. CRD42023427429). Registered on May 28, 2023

**Supplementary Information:**

The online version contains supplementary material available at 10.1038/s41598-025-28576-4.

## Introduction

Spinal infections (SI) encompass vertebral osteomyelitis (infection of the vertebral body), discitis (infection of the intervertebral disc), and spondylodiscitis (infection involving two adjacent vertebral bodies and their intervertebral disc) [1, 2]. The incidence of SI in developed nations ranges from 4 to 24 per million per year [3, 4], and has witnessed an upward trend in recent decades due to various factors, including a growing susceptible population (e.g., elderly individuals, immunocompromised patients, those with diabetes mellitus, chronic renal or liver diseases, and prolonged steroid usage); an increase in invasive procedures, particularly spinal surgeries; and advancements in diagnostic sensitivity [3]. Left untreated, SI can lead to debilitating complications such as structural spinal damage, neurological impairment, sepsis, and mortality [5–8].

SI is clinically diagnosed based on manifestations and imaging examinations, often resulting in a high misdiagnosis rate due to the lack of specificity [1, 9, 10]. The clinical manifestations of SI are atypical, resembling symptoms and signs of other conditions like spinal tumors and inflammatory diseases, leading to difficulties in differentiation [6, 11, 12]. Early changes in spinal structure can be detected through imaging examinations [1, 13], facilitating condition evaluation and providing essential support for early clinical interventions. Nonetheless, relying solely on imaging findings may lead to biased diagnoses [14, 15], making it challenging to differentiate between various types of SI. Therefore, there is a critical need for microbial diagnosis capable of identifying pathogenic microorganisms.

Microbial diagnosis involves the detection and identification of microorganisms in samples such as body fluids and tissues using techniques like culturing and molecular assays [16, 17], notably polymerase chain reaction (PCR). Microbial cultivation is considered the “gold standard” for diagnosing SI 18, facilitating the identification of microorganism types and their antimicrobial resistance profiles, thus guiding antibiotic use and improving clinical outcomes for SI patients. While tissue or blood culture is the most widely employed method, its time-consuming nature and low sensitivity are limitations [18–20]. Broad-spectrum molecular diagnostic approaches provide comprehensive information [17, 21] but are costly [22]. In contrast, PCR or multiplex polymerase chain reaction (MPCR) methods, although cost-effective, have limited capacity to diagnose multiple microorganisms. Understanding the infection spectrum aids in designing targeted primers, thereby enhancing the efficiency and accuracy of PCR and MPCR. Consequently, elucidating the infection spectrum is crucial for the application of PCR and MPCR.

Understanding the infection spectrum of SI plays a crucial role in guiding targeted testing and empirical antibiotic treatment for patients. By gaining insights into the prevalence of various pathogens, clinicians can anticipate potential types of pathogenic bacteria during the diagnostic process, enabling informed decisions regarding antibiotic selection. Additionally, exploring the infection spectrum facilitates the examination of risk factors, treatment efficacy, and prognosis associated with different pathogenic bacteria, thereby enhancing clinical treatment strategies [23]. Consequently, this article aims to conduct a meta-analysis to comprehensively determine the microbial spectrum of SI using blood culture, tissue culture, and molecular methods. The findings aim to provide a robust evidence base to inform empirical antibiotic therapy and guide the application of targeted diagnostic assays in clinical practice.

## Materials and methods

This study was registered in the International Prospective Register of Systematic Reviews database (CRD42023427429) and followed the Preferred Reporting Items for Systematic Review and Meta-Analyses (PRISMA) reporting guideline [24].

### Search strategy

We searched Embase, PubMed, and Web of Science using Medical Subject Headings (MeSH) terms and “search terms” (as listed in the Supplementary Methods). The most recent search was conducted on 2024/05/09. We applied no date or language restrictions, as detailed in supplementary Table S1.

### Study selection criteria

The inclusion criteria are as follows: (1) the participants are suspected cases of SI, including but not limited to spontaneous (hematogenous), post-surgical, and device-associated infections; (2) the detection method employed is blood culture, tissue culture, or molecular biology testing; (3) the study reports the types of pathogenic microorganisms and the number of infected individuals.

The exclusion criteria are as follows: (1) The molecular biology methods employed are other than 16S rRNA polymerase chain reaction or metagenomic next-generation sequencing (mNGS); (2) The sample type is non-blood and non-tissue; (3) Case reports with less than five participants; (4) Research published in the form of abstracts and reviews; (5) Research on detecting specific bacterial strains.

### Data extraction

The extracted materials include: (1) The author and year of publication; (2) The country conducting the research; (3) Population characteristics; (4) Sample type; (5) Pathogen type; (6) Pathogenic examination results (7) Detection method; (8) Related findings: number of positive cases. Two investigators (RZ and MDW) independently screened article titles and abstracts retrieved from the literature search. Full texts of potentially eligible studies were further assessed for final inclusion. A third investigator (GZZ) cross-checked extracted data, and disagreements were resolved through discussion and consensus.

### Quality assessment

The methodological quality of the included studies was assessed using the Joanna Briggs Institute (JBI) critical appraisal checklist for prevalence studies. Two independent reviewers (RZ and MDW) conducted the appraisal. To ensure consistency and objectivity, pre-defined operational criteria were established for each of the eight items on the checklist, as detailed in supplementary Table S2.

For instance, Item 3 (“Was the exposure measured in a valid and reliable way?”) was operationalized in the context of this review as pertaining to microbiological diagnostic methods. A rating of “Yes” was assigned only to studies that provided explicit descriptions of the techniques employed (e.g., culture conditions, PCR primers and protocols). Any disagreements between the two primary reviewers were resolved through discussion and consensus with a third reviewer (GZZ).

An overall quality score for each study was calculated by summing the number of “Yes” responses, yielding a score ranging from 0 to 8. Based on these scores, studies were categorized as high quality (scores 7–8), moderate quality (scores 4–6), or low quality (scores 0–3).

### Statistical analysis

A random-effects model was used to calculate pooled results and to estimate a 95% confidence interval (CI). The model employs the inverse-variance method to calculate weighted averages, and gives more weight to studies with larger sample sizes and greater precision. The *I*^*2*^ statistic was used to assess the heterogeneity of included studies, with *I*^*2*^ > 50% suggesting significant heterogeneity. All *P*-values were two-sided. A *P*-value < 0.05 was considered statistically significant. This meta-analysis was conducted using the “meta” package in R statistical software version 4.4.1.

## Result

### Study selection and characteristics

**Fig. 1:**
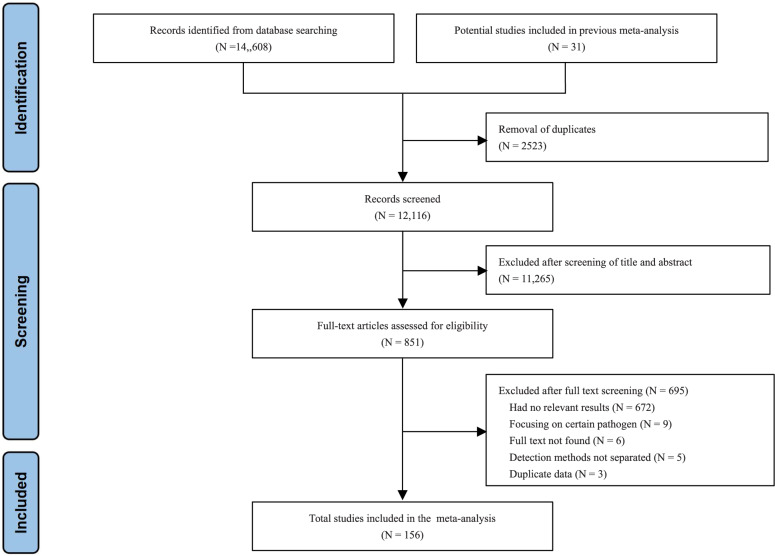
Study selection.

A total of 14,639 papers were retrieved. After excluding duplicate and irrelevant papers, a total of 156 studies, including 13,539 patients, were included. The literature retrieval flow chart is shown in Figure 1.

A total of 156 studies were included in the analysis, with 73 originating from Asia, 45 from Europe, 35 from North America, 1 from South America, and 2 from Africa. Furthermore, 50 studies employed blood culture, 135 studies employed tissue culture, 16 studies employed molecular biology detection methods, 27 studies employed blood and tissue culture, 2 studies employed tissue culture and molecular biology detection methods, and 8 studies employed all three methods. Sixty-eight studies reported on the number of patients presenting with fever. For a detailed overview of the characteristics of the studied individuals, please refer to supplementary Table S3.

The methodological quality assessment using the JBI checklist revealed that the included studies were predominantly of intermediate quality (n=120), with only 9 studies rated as high quality. This distribution is largely attributable to the performance on items related to confounding factors. Specifically, only 19.2% of studies identified potential confounders (Q5), and a mere 2.6% stated strategies to address them (Q6). It is critical to interpret these findings in the context of the included literature, which primarily consisted of case series. The descriptive aim of such studies does not typically necessitate the formal identification or control of confounders, which is a standard geared toward analytical study designs. This is further evidenced by the high proportion of studies (71.8%) for which item Q6 was deemed “Not Applicable”. Detailed information can be found in Table 1 and supplementary Table S4.

**Table 1:** Summary of JBI Critical Appraisal Checklist Results.

JBI critical appraisal checklist	Yes, n (%)	No, n (%)	Unclear, n (%)	Not Applicable, n (%)
1. Were the criteria for inclusion in the sample clearly defined?	82 (52.6%)	72 (46.2%)	2 (1.3%)	0 (0.0%)
2. Were the study subjects and the setting described in detail?	123 (78.8%)	33 (21.6%)	0 (0.0%)	0 (0.0%)
3. Was the exposure measured in a valid and reliable way?	149 (95.5%)	2 (1.3%)	5 (3.2%)	0 (0%)
4. Were objective, standard criteria used for measurement of the condition?	95 (60.9%)	9 (5.8%)	52 (33.3%)	0 (0.0%)
5. Were confounding factors identified?	30 (19.2%)	11 (7.1%)	115 (73.7%)	0 (0.0%)
6. Were strategies to deal with confounding factors stated?	4 (2.6%)	40 (25.6%)	0 (0.0%)	112 (71.8%)
7. Were the outcomes measured in a valid and reliable way?	156 (100.0%)	0 (0.0%)	0 (0.0%)	0 (0.0%)
8. Was appropriate statistical analysis used?	104 (66.7%)	19 (12.2%)	33 (21.2%)	0 (0.0%)

### Bacterial infection spectrum

#### Conventional culture methods

Conventional culture methods revealed a consistent profile of pathogens, with tissue culture generally yielding higher and broader detection rates. Gram-positive bacteria, particularly *staphylococci*, were the most prevalent. The pooled infection rate for this group was 24.0% (95%CI: 19.1–29.2) in blood culture and 26.3% (95%CI: 23.2–29.6) in tissue culture. Within the *staphylococci*, *Staphylococcus aureus* was the dominant pathogen, identified with pooled infection rates of 17.6% (95%CI: 12.8−22.9) and 16.8% (95%CI: 14.0−19.8) by blood and tissue culture, respectively. Tissue culture further characterized these isolates into methicillin-sensitive (MSSA, 6.7%) and methicillin-resistant (MRSA, 5.2%) strains. Notably, the detection rate for coagulase-negative staphylococci (CoNS) was significantly higher in tissue culture (6.4%) than in blood culture (3.5%), underscoring the value of tissue sampling in confirming true infection versus contamination. Among other gram-positive bacteria, *streptococci* were detected at rates of 3.9% (blood culture) and 4.0% (tissue culture), while *enterococci* were below 1%.

Among Gram-negative bacteria, *Enterobacterales* were the primary group detected, with *Escherichia coli* as the most prominent species. Its detection rate was higher in tissue culture (2.9%) than in blood culture (1.8%). The overall yield for Gram-negative rods was low, with *Pseudomonas aeruginosa* being detected primarily by tissue culture (0.5%). Tissue culture also proved capable of detecting specific pathogens that were rarely recovered from blood, including *Cutibacterium acnes* (0.7%) and MTB (1.3%), highlighting the superior comprehensiveness of tissue sampling.

#### Molecular biology methods

Molecular biology methods demonstrated unique diagnostic advantages, particularly for pathogens that are difficult to culture by conventional means. For common pyogenic bacteria, their detection rates differed from those of culture-based methods. For instance, the pooled infection rate for *Staphylococcus aureus* was 12.0% (95%CI: 9.3–15.0), lower than that from tissue culture, whereas the rate for *Streptococcus spp*. (6.1%, 95%CI: 3.7–9.1) was comparatively higher.

**Fig. 2:**
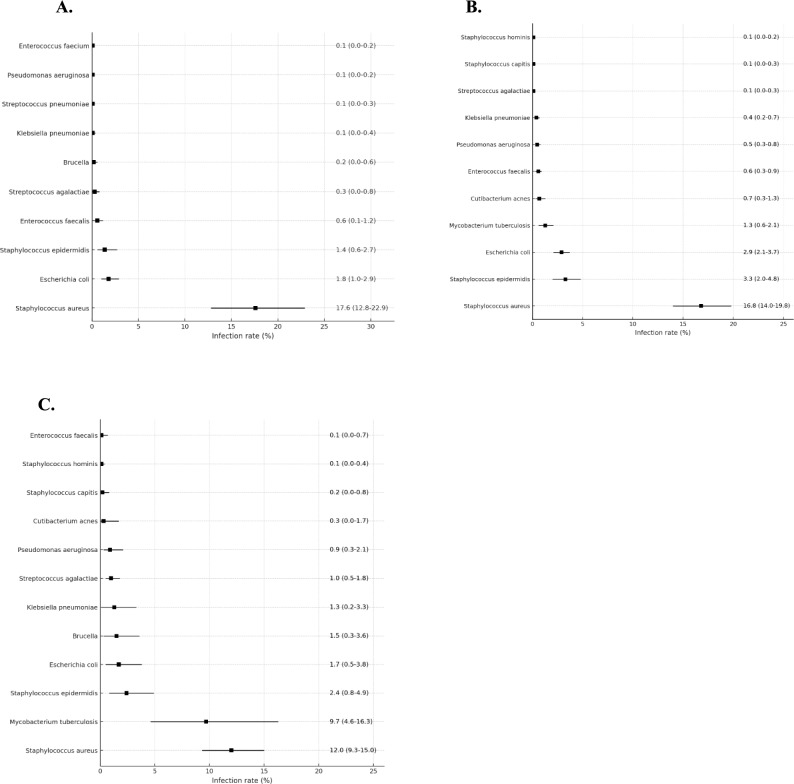
Bacterial infection rates determined by three diagnostic methods. **A**. Blood culture bacterial infection rate; **B**. Tissue culture bacterial infection rate; **C**. Molecular biology methods bacterial infection rate. Dots represent point estimates, and horizontal lines indicate 95% confidence intervals.

The most striking finding was the exceptionally high detection rate of MTB by molecular methods, with a pooled infection rate of 9.7% (95%CI: 4.6–16.3). This rate far exceeded those obtained by tissue culture (1.3%) and blood culture (0.0%), indicating the indispensable role of molecular diagnosis in confirming tuberculous spondylodiscitis. Similarly, for *Brucella*, the detection rate by molecular methods (1.5%) was higher than that by either blood or tissue culture. Additionally, molecular methods reported a high infection rate in the “Other pathogens” category (6.2%, 95%CI: 2.8–10.8), suggesting their ability to detect a wider array of atypical or difficult-to-culture microorganisms. Detailed information can be found in Table 2 and Figure 2.

**Table 2:** Microbial infection rates in blood culture, tissue culture, and molecular biology methods.

	Blood culture				Tissue culture				Molecular biology methods			
	Studies (n)	Individuals (n)	Infection rates (95% CI)	*I*^*2*^ (%)	Studies (n)	Individuals (n)	Infection rates (95% CI)	*I*^*2*^ (%)	Studies (n)	Individuals (n)	Infection rates (95% CI)	*I*^*2*^ (%)
Gram-Positive Bacteria												
*Staphylococci spp.*	46	3520	24.0 (19.1−29.2)	92	137	12061	26.3 (23.2−29.6)	96	17	910	20.5 (14.7−26.9)	75
*Staphylococcus aureus*	46	3520	17.6 (12.8−22.9)	93	137	12096	16.8 (14.0−19.8)	96	17	910	12.0 (9.3−15.0)	35
MSSA	16	1958	8.3 (3.8−14.2)	93	57	4797	6.7 (4.1−9.9)	93	-	-	-	-
MRSA	17	1998	4.1 (1.7−7.4)	83	61	4882	5.2 (3.5−7.2)	87	-	-	-	-
CoNS*	44	3475	3.5 (2.2−5.1)	79	132	11744	6.4 (4.9−8.1)	89	17	910	5.5 (1.9−10.9)	83
*Staphylococcus epidermidis*	32	1979	1.4 (0.6−2.7)	76	91	5447	3.3 (2.0−4.8)	85	16	888	2.4 (0.8−4.9)	68
*Staphylococcus hominis*	27	1465	0.1 (0.0−0.2)	0	77	3608	0.1 (0.0−0.2)	0	14	565	0.1 (0.0−0.4)	14
*Staphylococcus capitis*	26	1389	0.0 (0.0−0.1)	0	84	4768	0.1 (0.0−0.3)	0	14	565	0.2 (0.0−0.8)	18
Other *Staphylococci*	30	1854	0.2 (0.0−0.7)	33	91	5474	0.4 (0.2−0.7)	48	16	888	0.5 (0.0−1.6)	63
*Streptococcus spp.*	44	3470	3.9 (2.2−6.0)	87	137	12050	4.0 (3.1−4.9)	83	17	910	6.1 (3.7−9.1)	62
*Α-Hemolytic streptococcus*	33	2223	0.4 (0.1−1.1)	66	100	7783	0.3 (0.1−0.6)	64	16	883	0.3 (0.0−1.0)	31
*Streptococcus pneumoniae*	31	2171	0.1 (0.0−0.3)	0	82	6324	0.0 (0.0−0.1)	0	11	609	0.0 (0.0−0.3)	0
*Streptococcus agalactiae*	32	2086	0.3 (0.0−0.8)	54	100	7836	0.1 (0.0−0.3)	24	16	883	1.0 (0.5−1.8)	6
*Group D Streptococcus*	35	2324	0.8 (0.2−1.6)	68	113	9839	1.0 (0.6−1.3)	63	15	849	0.4 (0.0−1.1)	30
Other *Streptococcus*	36	2364	0.2 (0.0−0.5)	26	105	8442	0.4 (0.2−0.6)	45	16	883	1.8 (0.6−3.8)	59
*Enterococcus spp*	35	2277	0.7 (0.2−1.4)	64	102	7770	0.6 (0.2−0.8)	45	14	804	0.0 (0.0−0.5)	0
*Enterococcus faecalis*	35	2277	0.6 (0.1−1.2)	58	102	7770	0.6 (0.3−0.9)	47	14	804	0.1 (0.0−0.7)	0
*Enterococcus faecium*	33	2058	0.1 (0.0−0.2)	0	101	7918	0.0 (0.0−0.1)	0	13	785	0.0 (0.0−0.0)	0
*Cutibacterium acnes*	44	2968	0.0 (0.0−0.1)	0	120	10439	0.7 (0.3−1.3)	84	17	910	0.3 (0.0−1.7)	70
Gram-Negative Bacteria												
*Enterobacterales*	42	2778	3.0 (1.7−4.7)	76	129	10889	4.9 (3.9−6.2)	84	17	910	5.2 (2.4−9.0)	76
*Escherichia coli*	42	2778	1.8 (1.0−2.9)	63	126	8804	2.9 (2.1−3.7)	77	17	910	1.7 (0.5−3.8)	69
*Klebsiella pneumoniae*	37	1988	0.1 (0.0−0.4)	6	115	7529	0.4 (0.2−0.7)	52	17	910	1.3 (0.2−3.3)	74
*Proteus*	40	2443	0.1 (0.0−0.3)	0	118	7942	0.0 (0.0−0.1)	0	17	910	0.0 (0.0−0.3)	0
Other *Enterobacter*	41	2545	0.1 (0.0−0.2)	0	112	8358	0.1 (0.0−0.2)	0	17	910	0.3 (0.0−0.7)	0
*Brucella*	43	2804	0.2 (0.0−0.6)	62	121	10502	0.0 (0.0−0.1)	0	17	910	1.5 (0.3−3.6)	75
*Pseudomonas aeruginosa*	42	2702	0.1 (0.0−0.2)	0	119	9747	0.5 (0.3−0.8)	56	17	910	0.9 (0.3−2.1)	44
*Mycobacterium tuberculosis*	43	3361	0.0 (0.0−0.1)	0	122	11058	1.3 (0.6−2.1)	86	17	910	9.7 (4.6−16.3)	90
Fungus	44	3422	0.1 (0.0−0.2)	0	133	11786	0.3 (0.2−0.5)	43	17	910	0.7 (0.1−1.8)	48
Other pathogens	45	3510	0.9 (0.4−1.6)	66	137	12067	2.0 (1.4−2.8)	88	17	910	6.2 (2.8−10.8)	80

Note: *CoNS: Coagulase−negative staphylococcus.

### Subgroup analysis

Subgroup analyses identified several significant sources of heterogeneity. The detection rate of Mycobacterium tuberculosis by molecular assays was markedly higher in regions with a high TB burden (31–100/100,000) compared to low-burden regions (16.8% vs 1.2%, p=0.0001) and in studies published after 2010 (14.0% vs 0.3%, p=0.0021). For bacterial pathogens, study quality, sample size, and demographic factors did not consistently affect culture-based detection rates. However, an inverse association was observed between sample size and detection rate for molecular tests (e.g., for E. coli, >50 samples: 4.2% vs ≤50 samples: 0.8%, p=0.0523), suggesting a small-study effect. The brucellosis burden was a specific predictor for Brucella detection in blood culture (moderate: 1.0% vs low: 0.0%, p=0.0063), please refer to supplementary Table S5-22.

### Comparison between molecular biology methods and conventional culture

In our subgroup analysis of the six bacteria with the highest infection rates, we compared the detection rates of various pathogens using 16S rRNA PCR and mNGS against conventional culture. Notably, the detection rate of MTB using mNGS was 18.5% (95%CI: 13.6−23.9, *I*^*2*^ = 55%), which is significantly higher than the detection rate achieved by conventional culture, which was 2.8% (95%CI: 0.2−8.3,* I*^*2*^ = 90%). The odds ratio (OR) for mNGS detection relative to conventional culture was 4.24 (95%CI: 1.68−10.73,* I*^*2*^ = 57%). No significant difference was observed in the detection rates of other bacteria when comparing molecular biology methods to conventional culture techniques. Detailed information can be found in Table 3.

**Table 3:** Detection rate of pathogenic bacteria by 16 s rRNA PCR and mNGS and comparison with conventional culture.

	Studies (n)	Participants (n)	OR	*I*^*2*^ (%)
*Staphylococcus aureus*				
16 s rRNA PCR vs Culture	4	239	0.78 (0.44−1.39)	0
mNGS vs Culture	9	530	1.34 (0.89−2.03)	0
*Staphylococcus epidermidis*				
16 s rRNA PCR vs Culture	3	220	0.64 (0.21−1.92)	0
mNGS vs Culture	9	530	0.95 (0.37−2.47)	27
*Escherichia coli*				
16 s rRNA PCR vs Culture	4	239	1.48 (0.53−4.09)	0
mNGS vs Culture	8	422	1.13 (0.55−2.29)	0
*Klebsiella pneumonia*				
16 s rRNA PCR vs Culture	4	239	1.46 (0.22−9.49)	0
mNGS vs Culture	9	530	1.60 (0.78−3.30)	0
*Brucella*				
16 s rRNA PCR vs Culture	4	239	1.36 (0.25−7.38)	0
mNGS vs Culture	9	530	1.16 (0.68−2.00)	0
*Mycobacterium tuberculosis*				
16 s rRNA PCR vs Culture	4	239	1.00 (0.17−5.00)	0
mNGS vs Culture	9	530	4.24 (1.68−10.73)	57

## Discussion

This meta-analysis quantifies the bacterial spectrum of SI and the performance of key diagnostic methods. The microbial landscape is dominated by Gram-positive bacteria, principally *Staphylococcus aureus*. Our findings confirm a complementary diagnostic role for culture and molecular techniques: tissue culture provides the most balanced profile for pyogenic bacteria, while mNGS exhibits superior sensitivity specifically for MTB and *Brucella*. These results have direct implications for optimizing diagnostic pathways and empirical therapy in SI.

Our findings support Gram-positive bacteria as the principal pathogens in SI, with *Staphylococcus aureus* demonstrating the highest detection rate across all diagnostic methods. Its high prevalence is closely linked to key virulence factors, including robust invasive capacity and a pronounced ability to form biofilms on bone and implant surfaces [25, 26], which collectively facilitate the establishment of persistent infections. Furthermore, as a common cause of skin and soft tissue infections [27, 28], *S. aureus* is well-positioned to seed the spine following surgery or trauma. Of particular clinical relevance, methicillin-resistant *S. aureus* (MRSA) accounts for a substantial proportion of cases (5.2% detection rate in tissue culture). Consequently, pending definitive microbiological results, empiric antibiotic therapy for SI—particularly in post-surgical or post-traumatic settings—should prioritize coverage against *S. aureus* and ensure activity against both methicillin-sensitive and methicillin-resistant strains. This approach is likely critical to the success of initial treatment.

Beyond *Staphylococcus aureus*, the diagnosis and management of spinal infections are complicated by two common Gram-positive organisms: *Staphylococcus epidermidis* and *Cutibacterium acnes*. As both are frequent skin commensals, accurately distinguishing true infection from contamination remains a major clinical challenge. The isolation of *S. epidermidis* from tissue cultures in 3.3% of cases highlights its relevance in spinal infection. Its pathogenicity is largely driven by biofilm formation on implanted devices, mediated by polysaccharide adhesins that enable persistent colonization [29]. In contrast, *C. acnes* typically causes indolent, delayed-onset infections. Introduced during surgery, this slow-growing anaerobic bacterium can remain dormant for weeks to months before symptoms emerge [30]. Therefore, to improve microbiological yield and establish a reliable diagnosis, it is essential to collect multiple deep-tissue specimens intraoperatively and to specifically request extended anaerobic culture incubation—up to 10–14 days—to facilitate the detection of *C. acnes *[31].

Among Gram-negative bacteria, *Escherichia coli* had the highest infection rate (2.9% detected by tissue culture). Studies indicate that patients with a history of urinary tract infections, recent abdominal surgery, or immunocompromised status are at significantly increased risk of hematogenous spread of *E. coli* to the spine, representing a key route of infection [32]. Of particular concern is *Pseudomonas aeruginosa*. Although its overall detection rate in tissue culture was only 0.5%, its intrinsic resistance to multiple antibiotics renders it clinically formidable [33]. Infections with this pathogen are frequently associated with healthcare exposure and intravenous drug use [34]. Consequently, for patients with these risk factors, empiric therapy covering *P. aeruginosa* is recommended pending susceptibility results. Suitable options include anti-pseudomonal cephalosporins (e.g., ceftazidime, cefepime), carbapenems, or piperacillin-tazobactam.

We systematically compared the diagnostic performance of three detection methods for the pathogenic diagnosis of SI, revealing their distinct and complementary roles. A key finding is the marked advantage of molecular biological methods, particularly mNGS, in detecting difficult-to-culture pathogens such as MTB. The detection rate of MTB by mNGS reached 9.7%, and the likelihood of a positive detection was 4.24 times higher than that of traditional culture. This discrepancy stems primarily from the slow growth and fastidious culture requirements of MTB, which inherently lead to delays and potential missed diagnoses with culture-dependent methods [35, 36]. In contrast, mNGS enables rapid and precise identification of MTB by directly detecting pathogen nucleic acids [37, 38]. Regarding *Brucella*, although the direct comparison did not reach statistical significance, pooled data indicated a markedly higher detection rate by molecular biology methods (1.5%) compared to traditional culture (0.0–0.2%), strongly suggesting the significant potential of molecular methods for diagnosing such specific infections.

Based on these findings, a tiered and optimized diagnostic pathway is recommended to enhance diagnostic efficiency, particularly in light of the substantial proportion of culture-negative cases which suggests a significant prevalence of “sterile” or difficult-to-culture spinal infections. First, when the clinical differential diagnosis includes MTB, mNGS should be considered a first-line diagnostic tool alongside tissue culture. This is particularly advised for patients from endemic areas or with suggestive epidemiological histories; simultaneous testing during initial surgery can circumvent the low sensitivity and prolonged turnaround time of traditional culture, providing critical evidence for early targeted therapy. Second, for the challenging scenario of culture-negative infection—where routine bacterial culture (e.g., for *Staphylococcus aureus*) fails to yield a pathogen— molecular testing should be incorporated as an essential supplementary step to uncover fastidious or prior-antibiotic-affected pathogens. It is important to note that broad-spectrum molecular methods like mNGS and 16S rRNA sequencing are relatively expensive; thus, PCR or multiplex PCR represent viable and more cost-effective alternatives in certain scenarios. In summary, an intelligent combination of cost-effective traditional culture—which remains indispensable for providing antimicrobial susceptibility testing—and highly sensitive molecular technologies offers an optimal balance among rapid diagnosis, precise treatment, and cost control for both culture-positive and culture-negative spinal infections.

Our study has several limitations. Firstly, while our analysis pooled data from all types of SI, it did not allow for a detailed subgroup analysis of these distinct clinical entities. Consequently, the presented summary estimates may not fully represent any specific clinical context. Second, a key methodological constraint is our inability to account for the analytical sensitivity of the diagnostic tests used in the primary studies. This includes the unknown impact of prior antibiotic use (which we could not systematically ascertain) and the undefined lower limit of detection for cultures and molecular assays. Consequently, negative results primarily indicate that the pathogen load was below the test’s detection threshold, not absolute sterility. Thirdly, almost all of the included studies are retrospective observational studies. Finally, despite conducting extensive subgroup analyses and meta-regressions that identified several significant sources of heterogeneity (e.g., geographical disease burden for specific pathogens), a substantial portion of heterogeneity remained unexplained.

## Conclusion

This review confirms that a core group of pathogens, including Staphylococcus *aureus, Staphylococcus epidermidis*, MTB, and *Escherichia coli*. Our findings underscore that tissue culture is fundamental for common pyogenic bacteria, while metagenomic next-generation sequencing is indispensable for detecting fastidious organisms like MTB.

## Supplementary Information

Supplementary Information 1.

Supplementary Information 2.

## Data Availability

All data generated or analysed during this study are included in this published article.

## Abbreviations

SI: Spinal infections
PCR: Polymerase chain reaction
MPCR: Multiplex polymerase chain reaction
*S. aureus*: *Staphylococcus aureus*
MTB: Mycobacterium tuberculosis
mNGS: Metagenomic next-generation sequencing

## References

[1] Lazzeri, E. et al. Joint EANM/ESNR and ESCMID-endorsed consensus document for the diagnosis of spine infection (spondylodiscitis) in adults. *Eur. J. Nucl. Med. Mol. Imaging* **46**(12), 2464–2487 (2019). 31399800 10.1007/s00259-019-04393-6

[2] Calderone, R. R. & Larsen, J. M. Overview and classification of spinal infections. *Orthop. Clin. North Am.* **27**(1), 1–8 (1996). 8539040

[3] Gouliouris, T., Aliyu, S. H. & Brown, N. M. Spondylodiscitis: Update on diagnosis and management. *J. Antimicrob. Chemother.* **65**, 311–324 (2010). 10.1093/jac/dkq303 20876624

[4] Gasbarrini, A. L. et al. Clinical features, diagnostic and therapeutic approaches to haematogenous vertebral osteomyelitis. *Eur. Rev. Med. Pharmacol. Sci.* **9**(1), 53–66 (2005). 15852519

[5] Kwon, J. W., Hyun, S. J., Han, S. H., Kim, K. J. & Jahng, T. A. Pyogenic vertebral osteomyelitis: Clinical features, diagnosis, and treatment. *Korean J. Spine.* **14**(2), 27–34 (2017). 28704905 10.14245/kjs.2017.14.2.27 PMC5518432

[6] Skaf, G. S. et al. Pyogenic spondylodiscitis: an overview. *J. Infect. Public Health* **3**(1), 5–16 (2010). 20701886 10.1016/j.jiph.2010.01.001

[7] Kim, J., Oh, S. H., Kim, S. W. & Kim, T. H. The epidemiology of concurrent infection in patients with pyogenic spine infection and its association with early mortality: A nationwide cohort study based on 10,695 patients. *J. Infect. Public Health.* **16**(6), 981–988 (2023). 37148755 10.1016/j.jiph.2023.04.010

[8] Papachristodoulou, E., Kakoullis, L., Louppides, S. & Panos, G. Granulomatous infective spondylitis in a patient presenting with progressive difficulty in walking: The differential between tuberculosis and brucellosis. *BMJ Case Rep.* **12**(11), e232540 (2019). 31767612 10.1136/bcr-2019-232540 PMC6887356

[9] Chen, X., Ye, J., Lei, H. & Wang, C. Novel potential diagnostic serum biomarkers of metabolomics in osteoarticular tuberculosis patients: A preliminary study. *Front. Cell Infect. Microbiol.* **12**, 827528 (2022). 35402287 10.3389/fcimb.2022.827528 PMC8992656

[10] Tali, E. T., Oner, A. Y. & Koc, A. M. Pyogenic spinal infections. *Neuroimaging Clin. N. Am.* **25**(2), 193–208 (2015). 25952173 10.1016/j.nic.2015.01.003

[11] Hetem, S. F. & Schils, J. P. Imaging of infections and inflammatory conditions of the spine. *Semin. Musculoskelet. Radiol.* **4**(3), 329–347. 10.1055/s-2000-9342 (2000). 11371323 10.1055/s-2000-9342

[12] Rutges, J. P., Kempen, D. H., van Dijk, M. & Oner, F. C. Outcome of conservative and surgical treatment of pyogenic spondylodiscitis: A systematic literature review. *Eur. Spine J.* **25**(4), 983–999 (2016). 26585975 10.1007/s00586-015-4318-y

[13] Cheung, W. Y. & Luk, K. D. Pyogenic spondylitis. *Int. Orthop.* **36**(2), 397–404 (2012). 22033610 10.1007/s00264-011-1384-6 PMC3282872

[14] Raghavan, M., Lazzeri, E. & Palestro, C. J. Imaging of Spondylodiscitis. *Semin. Nucl. Med.* **48**(2), 131–147 (2018). 29452617 10.1053/j.semnuclmed.2017.11.001

[15] Leone, A. et al. Imaging of spondylodiscitis. *Eur. Rev. Med. Pharmacol. Sci.* **16**(Suppl 2), 8–19 (2012). 22655479

[16] Both, A. et al. The added value of a commercial 16S/18S-PCR assay (UMD-SelectNA, Molzym) for microbiological diagnosis of spondylodiscitis: An observational study. *Diagn. Microbiol. Infect. Dis.* **106**(1), 115926 (2023). 36963329 10.1016/j.diagmicrobio.2023.115926

[17] Fuursted, K., Arpi, M., Lindblad, B. E. & Pedersen, L. N. Broad-range PCR as a supplement to culture for detection of bacterial pathogens in patients with a clinically diagnosed spinal infection. *Scand. J. Infect. Dis.* **40**(10), 772–777 (2008). 18609207 10.1080/00365540802119994

[18] Sheikh, A. F. et al. Pathogen identification in suspected cases of pyogenic spondylodiscitis. *Front. Cell Infect. Microbiol.* **7**, 60 (2017). 28337426 10.3389/fcimb.2017.00060 PMC5343039

[19] Huang, H. et al. Pathogen detection in suspected spinal infection: metagenomic next-generation sequencing versus culture. *Eur. Spine J.* **32**(12), 4220–4228 (2023). 37237239 10.1007/s00586-023-07707-3

[20] Ma, C. et al. The potential of metagenomic next-generation sequencing in diagnosis of spinal infection: A retrospective study. *Eur. Spine J.* **31**(2), 442–447 (2022). 34677679 10.1007/s00586-021-07026-5

[21] Wang, G. et al. Application of metagenomic next-generation sequencing in the detection of pathogens in spinal infections. *Spine J.* **23**(6), 859–867 (2023). 36773890 10.1016/j.spinee.2023.02.001

[22] Diao, Z., Han, D., Zhang, R. & Li, J. Metagenomics next-generation sequencing tests take the stage in the diagnosis of lower respiratory tract infections. *J. Adv. Res.* **38**, 201–212 (2022). 35572406 10.1016/j.jare.2021.09.012 PMC9091713

[23] Zhu, Y. et al. Bacterial spectrum analysis and antimicrobial susceptibility study of osteoradionecrosis of the jaw in Southern China. *Oral Dis.* **28**(7), 2015–2025 (2022). 34273905 10.1111/odi.13968

[24] Page, M. J. et al. The PRISMA 2020 statement: an updated guideline for reporting systematic reviews. *BMJ* **372**, n71 (2021). 33782057 10.1136/bmj.n71 PMC8005924

[25] Ramirez, A. M. et al. SarA plays a predominant role in controlling the production of extracellular proteases in the diverse clinical isolates of Staphylococcus aureus LAC and UAMS-1. *Virulence* **11**(1), 1738–1762 (2020). 33258416 10.1080/21505594.2020.1855923 PMC7738309

[26] Sherman, E., Bayles, K., Moormeier, D., Endres, J. & Wei, T. Observations of shear stress effects on staphylococcus aureus biofilm formation. *mSphere* **4**(4), e00372-e419 (2019). 31315967 10.1128/mSphere.00372-19 PMC6637047

[27] Visvabharathy, L. et al. Group 1 CD1-restricted T cells contribute to control of systemic Staphylococcus aureus infection. *PLoS Pathog.* **16**(4), e1008443 (2020). 32343740 10.1371/journal.ppat.1008443 PMC7188215

[28] Cheung, G., Bae, J. S. & Otto, M. Pathogenicity and virulence of Staphylococcus aureus. *Virulence* **12**(1), 547–569 (2021). 33522395 10.1080/21505594.2021.1878688 PMC7872022

[29] Kouijzer, I. J. E. et al. Treatment and follow-up of vascular graft and endograft infection: Delphi consensus document. *Clin. Microbiol. Infect.*10.1016/j.cmi.2025.07.020 (2025). 40744275 10.1016/j.cmi.2025.07.020

[30] Huang, T. Y., Jiang, Y. E. & Scott, D. A. Culturable bacteria in the entire acne lesion and short-chain fatty acid metabolites of Cutibacterium acnes and Staphylococcus epidermidis isolates. *Biochem. Biophys. Res. Commun.* **24**(622), 45–49 (2022). 10.1016/j.bbrc.2022.06.068 35843093

[31] Werner, A. et al. Diagnostic value of preoperative joint aspiration for periprosthetic shoulder infection: Analysis of microbiological aspects and preoperative ICM minor criteria. *Arch. Orthop. Trauma Surg.* **145**(1), 413 (2025). 40833468 10.1007/s00402-025-06032-2

[32] Mo, Y. F. et al. Surgery combined with antibiotics for thoracic vertebral Escherichia coli infection after acupuncture: A case report. *World J. Clin. Cases* **10**(35), 13099–13107 (2022). 36569001 10.12998/wjcc.v10.i35.13099 PMC9782942

[33] Culotti, A. & Packman, A. I. Pseudomonas aeruginosa promotes Escherichia coli biofilm formation in nutrient-limited medium. *PLoS ONE* **9**(9), e107186 (2014). 25198725 10.1371/journal.pone.0107186 PMC4157881

[34] Yuan, Y. et al. Extracellular products-mediated interspecific interaction between Pseudomonas aeruginosa and Escherichia coli. *J. Microbiol.* **59**(1), 29–40 (2021). 33355890 10.1007/s12275-021-0478-0

[35] Lama, C. et al. Evaluation of Xpert MTB/RIF Assay, MTB culture and line probe assay for the detection of MDR Tuberculosis in AFB Smear negative specimens. *Diseases* **10**(4), 82 (2022). 36278581 10.3390/diseases10040082 PMC9624312

[36] Kanade, S., Mohammed, Z., Kulkarni, A. & Nataraj, G. Comparison of xpert MTB/RIF assay, line probe assay, and culture in diagnosis of pulmonary tuberculosis on bronchoscopic specimen. *Int. J. Mycobacteriol.* **12**(2), 151–156 (2023). 37338476 10.4103/ijmy.ijmy_86_23

[37] Chen, Y., Tang, H., Zheng, J., Yang, Q. & Han, D. Transforming tuberculosis diagnosis with clinical metagenomics: Progress and roadblocks. *J. Clin. Microbiol.* **19**, e0053725 (2025). 10.1128/jcm.00537-25 PMC12710363 40970697

[38] Li, Y. et al. Optimization of decision thresholds for Mycobacterium tuberculosis can effectively improve the performance of mNGS in tuberculosis diagnosis. *Front. Cell Infect. Microbiol.* **11**(15), 1646194 (2025). 10.3389/fcimb.2025.1646194 PMC12485630 41040987

