# Supplementary material for: The bacterial spectrum of spinal infections based on blood culture, tissue culture, and molecular methods: a systematic review and meta-analysis
Source: Sci Rep. 2025 Nov 28;15:45566. doi: 10.1038/s41598-025-28576-4 (PMC12753679; doi:10.1038/s41598-025-28576-4)
Supplement: Supplementary file 1 — Supplementary Information 1. [file 41598_2025_28576_MOESM1_ESM.docx]

**Supplementary web appendix for:**

**The Bacterial Spectrum of Spinal Infections Based on Blood Culture, Tissue Culture, and Molecular Methods: A Systematic Review and Meta-Analysis**

**Content**

[Table S1. Search strategy. 2](#_Toc29988)

[Table S2. Operational criteria for the Joanna Briggs Institute (JBI) critical appraisal tool 3](#_Toc24579)

[Table S3. Detailed characteristics of individual study. 4](#_Toc5436)

[Table S4. JBI critical appraisal checklist 13](#_Toc27649)

[Table S5 Subgroup analyses of blood culture assays for the detection of](#_Toc15507) *[Staphylococcus aureus](#_Toc15507)* [21](#_Toc15507)

[Table S6 Subgroup analyses of tissue-based assays for the detection of](#_Toc25518) *[Staphylococcus aureus](#_Toc25518)* [22](#_Toc25518)

[Table S7. Subgroup analyses of molecular assays for the detection of](#_Toc7575) *[Staphylococcus aureus](#_Toc7575)* [23](#_Toc7575)

[Table S8 Subgroup analyses of blood culture assays for the detection of](#_Toc13602) *[Mycobacterium tuberculosis](#_Toc13602)* [24](#_Toc13602)

[Table S9 Subgroup analyses of tissue-based assays for the detection of](#_Toc26073) *[Mycobacterium tuberculosis](#_Toc26073)* [25](#_Toc26073)

[Table S10. Subgroup analyses of molecular assays for the detection of](#_Toc22556) *[Mycobacterium tuberculosis](#_Toc22556)* [26](#_Toc22556)

[Table S11 Subgroup analyses of blood culture assays for the detection of](#_Toc29899) *[Brucella](#_Toc29899)* [27](#_Toc29899)

[Table S12 Subgroup analyses of tissue-based assays for the detection of](#_Toc586) *[Brucella](#_Toc586)* [28](#_Toc586)

[Table S13 Subgroup analyses of molecular assays for the detection of](#_Toc13658) *[Brucella](#_Toc13658)* [29](#_Toc13658)

[Table S14 Subgroup analyses of blood culture assays for the detection of](#_Toc14068) *[Escherichia coli](#_Toc14068)* [30](#_Toc14068)

[Table S15 Subgroup analyses of tissue-based assays for the detection of](#_Toc6519) *[Escherichia coli](#_Toc6519)* [31](#_Toc6519)

[Table S16 Subgroup analyses of molecular assays for the detection of](#_Toc21739) *[Escherichia coli](#_Toc21739)* [32](#_Toc21739)

[Table S17 Subgroup analyses of blood culture assays for the detection of](#_Toc28364) *[Klebsiella pneumoniae](#_Toc28364)* [33](#_Toc28364)

[Table S18 Subgroup analyses of the diagnostic performance of tissue-based assays for](#_Toc7391) *[Klebsiella pneumoniae](#_Toc7391)* [34](#_Toc7391)

[Table S19 Subgroup analyses of molecular assays for the detection of](#_Toc15920) *[Klebsiella pneumoniae](#_Toc15920)* [35](#_Toc15920)

[Table S20 Subgroup analyses of molecular assays for the detection of](#_Toc15252) *[Pseudomonas aeruginosa](#_Toc15252)* [36](#_Toc15252)

[Table S21 Subgroup analyses of blood culture assays for the detection of](#_Toc13201) *[Pseudomonas aeruginosa](#_Toc13201)* [37](#_Toc13201)

[Table S22 Subgroup analyses of tissue-based assays for the detection of](#_Toc32396) *[Pseudomonas aeruginosa](#_Toc32396)* [38](#_Toc32396)

**Table S1. Search strategy.**

| **Search strategy in PubMed** | | | | | |
| --- | --- | --- | --- | --- | --- |
| #1 | | | (((((((spinal infection) OR (spondylodiscitis)) OR (spondylitis)) OR (diskitis)) OR (vertebral osteomyelitis)) OR (spondylodiskitis)) OR (epidural abscess)) OR (paravertebral infection) | | 86,087 |
| #2 | | | (((((((((("Blood Culture"[Mesh]) OR (Blood Cultures)) OR (Culture, Blood)) OR (Blood Culture Test)) OR (Culture Test, Blood)) OR (((("Tissue Culture Techniques"[Mesh]) OR (Culture Techniques, Tissue)) OR (Tissue Culture Technique)) OR (Culture Technique, Tissue) OR Culture)) OR (((("Multiplex Polymerase Chain Reaction"[Mesh]) OR (Multiplex PCR)) OR (PCR, Multiplex)) OR (Multiplex Ligation-Dependent Probe Amplification))) OR (((("RNA, Ribosomal, 16S"[Mesh]) OR (16S rRNA)) OR (rRNA, 16S)) OR (16S))) OR ((((("Metagenomics"[Mesh]) OR (Community Genomics)) OR (Environmental Genomics)) OR (metagenomic Next-Generation Sequencing)) OR (mNGS))) OR ((((("Microarray Analysis"[Mesh]) OR (Microarray Microchip)) OR (Microarray Microchips)) OR (microbial microarrays)) OR (microbial chips))) | | 2,532,788 |
| #3 | | | #1 AND #2 | | 2,340 |
| **Search strategy in Embase** | | | | | |
| #1 | 'spinal infection'/exp OR 'spinal infection' OR (spinal AND ('infection'/exp OR infection)) OR spondylodiscitis OR spondylitis OR diskitis OR (vertebral AND osteomyelitis) OR spondylodiskitis OR (epidural AND abscess) OR (paravertebral AND infection) | | 120,495 | | |
| #2 | ('microarray Analysis'/exp OR (microarray AND microchip) OR (microbial AND microarrays) OR (microbial AND chips) OR 'tissue culture'/exp OR (tissue AND culture AND technique) OR (tissue AND culture) OR (blood AND culture) OR culture OR 'multiplex polymerase chain reaction'/exp OR (multiplex AND polymerase AND chain AND reaction) OR (multiplex AND ligation AND dependent AND probe AND amplification) OR 'metagenomic next generation sequencing'/exp OR 'metagenomic next generation sequencing' OR mngs OR 'RNA 16s'/exp OR (RNA, AND 16s) OR 16s) | | 2,148,619 | | |
| #3 | #1 AND #2 | | 5,110 | | |
| **Search strategy in Web-of-science** | | | | | |
| #1 | | | (((((((spinal infection) OR (spondylodiscitis)) OR (spondylitis)) OR (diskitis)) OR (vertebral osteomyelitis)) OR (spondylodiskitis)) OR (epidural abscess)) OR (paravertebral infection) | | 128,005 |
| #2 | | | (((((((("Blood Culture"[Mesh]) OR (Blood Cultures)) OR (Tissue Culture)) OR (Culture)) OR ((("Microarray Analysis"[Mesh]) OR (microbial chips)) OR (microbial microarrays))) OR ((("Metagenomics"[Mesh]) OR (metagenomic Next-Generation Sequencing)) OR (mNGS))) OR (("RNA, Ribosomal, 16S"[Mesh]) OR (16S rRNA))) OR (("Multiplex Polymerase Chain Reaction"[Mesh]) OR (Multiplex PCR))) | | 5,347,701 |
| #3 | | | #1 AND #2 | | 7,189 |

**Table S2. Operational criteria for the Joanna Briggs Institute (JBI) critical appraisal tool**

| **JBI Item** | **Question** | **Criteria for 'Y' (Yes)** | **Criteria for 'N' (No) / 'U' (Unclear) / 'NA' (Not Applicable)** |
| --- | --- | --- | --- |
| **Q1** | Were the criteria for inclusion in the sample clearly defined? | The study explicitly defined the diagnostic criteria for confirmed or suspected spinal infection (e.g., based on a combiNStion of clinical symptoms, imaging findings, microbiological, or histological results). | **N:** Mentioned "spinal infection patients" but provided no specific diagnostic criteria. **U:** The information was ambiguous and could not be clearly judged. **N**A: Not applicable. |
| **Q2** | Were the study subjects and the setting described in detail? | The study described basic demographic characteristics of the participants (e.g., mean age, sex distribution) and details of the clinical setting (e.g., tertiary hospital, study period, single/multi-center). | **N:** Description of the study population and setting was severely lacking. **U:** Description was incomplete. **N**A: Not applicable. |
| **Q3** | Was the exposure measured in a valid and reliable way? (Operationalized as: microbiological diagnostic methods) | The study provided explicit descriptions of the microbiological methods used (e.g., sample type, culture conditions and duration, PCR primers and targets, sequencing platforms). | **N:** Only vague statements were provided (e.g., "bacterial culture was performed") without methodological details. **U:** The methods for pathogen detection were not mentioned at all. **N**A: Not applicable. |
| **Q4** | Were objective, standard criteria used for measurement of the condition? | The study used and described objective criteria for confirming spinal infection (e.g., specific MRI/CT diagnostic findings, histopathological evidence, or clinical diagnostic guidelines). | **N:** Diagnosis was based solely on subjective clinical symptoms without objective criteria. **U:** The criteria used for confirmation were not specified. **N**A: Not applicable. |
| **Q5** | Were confounding factors identified? | The study identified or discussed potential confounding factors (e.g., prior antibiotic use, comorbidities like diabetes or immunosuppression, previous spinal surgery) that could affect pathogen detection rates. | **N:** No potential confounding factors were mentioned. **U:** Relevant factors were mentioned but not explicitly identified as confounders. **N**A: Not applicable. |
| **Q6** | Were strategies to deal with confounding factors stated? | The study employed specific strategies to control for confounders, either in the study design (e.g., matching) or statistical Analysis (e.g., multivariate regression). | **N:** Confounders were identified but no strategies to address them were stated. **U:** The strategy was described unclearly. **N**A: Not applicable. |
| Q7 | Were the outcomes measured in a valid and reliable way? (Operationalized as: pathogen detection outcome) | The primary outcome (pathogen detection) was defined clearly and consistently, with criteria to distinguish true infection from contamination or colonization. | N: The outcome definition was vague or missing. U: It was not described how the outcome was adjudicated. **N**A: Not applicable. |
| **Q8** | Was appropriate statistical Analysis used? | The study used correct descriptive statistics (e.g., rates, percentages, confidence intervals) and, if applicable, appropriate comparative tests (e.g., chi-square, t-test). | **N:** Statistical methods were used in error or were completely absent. **U:** The statistical methods used were not detailed. **N**A: Not applicable. |

**Table S3. Detailed characteristics of individual study**

| Study | Year | Study period | Country | Sample size | Age range (year) | Age median/mean | Male (%) | Study design | Fever (n) | Bacterial detection methods |
| --- | --- | --- | --- | --- | --- | --- | --- | --- | --- | --- |
| Ahl et al 1999[1] | 1999 | 1993.12−1996.11 | Sweden | 10 | 27−81 | 65.5 ± 16.64 | 50 | Case series | NS | Blood culture |
| Chandrasenan et al 2011[2] | 2011 | 2000−2007 | Czechia | 16 | 0.5−13 | 3.3 (mean) | 44 | Case series | 6 | Blood culture |
| Chen et al 2020[3] | 2020 | 2013.08−2017.05 | USA | 10 | 51−75 | 64.8 ± 8.08 | 50 | Case series | 3 | Blood culture |
| Colip et al 2018[4] | 2018 | 2010.01−2014.07 | USA | 167 | 20−72 | NS | NS | Case series | NS | Blood culture |
| Dholoo et al 2023[5] | 2023 | 2017.01−2019.10 | UK | 38 | 35−90 | NS | NS | Case series | 4 | Blood culture |
| Dobran et al 2017[6] | 2017 | 2011.01−2015.12 | Italy | 16 | 37−82 | 60.3 ± 14.9 | 56 | Case series | 5 | Blood culture |
| Guo et al 2021[7] | 2021 | 2006.12−2014.04 | China | 76 | 19−85 | NS | 70 | Case series | 11 | Blood culture |
| Jean et al 2017[8] | 2017 | 2006.11−2011.11 | France | 88 | 26−95 | 64.1 (mean) | 65 | RCT | 46 | Blood culture |
| Nakamura et al 2018[9] | 2018 | 2000−2014 | Japan | 126 | NS | 72  ±  11 | 55 | Cohort study | NS | Blood culture |
| Rankine et al 2004[10] | 2004 | NS | UK | 20 | 17M−78Y | 53 (mean) | 65 | Case series | NS | Blood culture |
| Rocha et al 2015[11] | 2015 | 2005.01−2011.12 | Portugal | 13 | 41−85 | 63.4 (mean) | 54 | Case series | 5 | Blood culture |
| Tang et al 2022[12] | 2022 | 2014.01−2016.12 | China | 37 | NS | 40.71  ±  17.04 | 43 | Cohort study | 24 | Blood culture |
| Wang et al 2012[13] | 2012 | 2004.01−2008.12 | Canada | 102 | 25−83 | 49.5 (mean) | 59 | Cohort study | NS | Blood culture |
| Zhang et al 2023[14] | 2023 | 2009.06−2019.06 | China | 6 | 20−61 | 33.7 (mean) | 6 | Case series | 2 | Blood culture |
| Ratiu et al 2024[15] | 2024 | 2013−2023 | USA | 32 | NS | 65 (mean) | 26 | Case control | 11 | Blood culture |
| Tal et al 2020[16] | 2020 | 2014−2019 | Israel | 12 | 36−80 | NS | 50 | Case series | NS | Tissue culture |
| Albert et al 2013[17] | 2013 | NS | UK | 61 | 18−65 | 46.4 ± 9.7 | 73 | Case series | NS | Tissue culture |
| Ameri et al 2021[18] | 2021 | 2006−2019 | USA | 31 | NS | 11.4 ± 2.3 | 16 | Case series | 2 | Tissue culture |
| Arya et al 1996[19] | 1996 | 1994.11−1996.01 | USA | 15 | 33−65 | 43.5 ± 2.24 | 67 | Case series | NS | Tissue culture |
| Avenel et al 2021[20] | 2021 | 2000.01−2009.12 | France | 300 | 48−75 | 63 (mean) | 59 | Cohort study | 88 | Tissue culture |
| Braun et al 2019[21] | 2019 | 2015.06−2018.03 | Germany | 40 | 10−89 | 65.1 (mean) | NS | Case series | NS | Tissue culture |
| Bürger et al 2019[22] | 2019 | 2016.09−2018.03 | Germany | 118 | NS | 62.3  ±  21.4 | 39 | Non-randomized | NS | Tissue culture |
| Burkhard et al 2021[23] | 2021 | 2014−2019 | Switzerland | 128 | 19−86 | 65.2 ± 14.8 | 55 | Case series | NS | Tissue culture |
| Cannavale et al 2022[24] | 2022 | 2016.01−2021.05 | Italy | 60 | NS | 59.2  ±  29 | 80 | Case series | 20 | Tissue culture |
| Callanan et al 2021[25] | 2021 | 2014.06−2016.06 | USA | 50 | 18−90 | 61.07 ± 15.1 | NS | Case series | NS | Tissue culture |
| Carlson et al 2020[26] | 2020 | 2007.06−2016.02 | USA | 152 | 14−84 | NS | 51 | Case series | NS | Tissue culture |
| Chang et al 2015[27] | 2015 | 2002.01−2011.12 | USA | 102 | 15−90 | 59 ± 17 | 51 | Case series | NS | Tissue culture |
| Chen et al 2015[28] | 2015 | 2006.10−2014.03 | China | 13 | 49−84 | 65.6 ± 9.73 | 38 | Case series | NS | Tissue culture |
| Chen et al 2021[29] | 2021 | 2017.05−2019.10 | China | 51 | 34−75 | 57.4 (mean) | 55 | Case series | 5 | Tissue culture |
| Choe et al 2014[30] | 2014 | NS | Japan | 32 | 37−85 | 69.6 ± 11.0 | 56 | RCT | 32 | Tissue culture |
| Choi et al 2010[31] | 2010 | 2003.05−2007.06 | South Korea | 9 | 21−61 | 39.7 ± 4.2 | 78 | Case series | 4 | Tissue culture |
| Cui et al 2022[32] | 2022 | 2018.10−2021.03 | China | 24 | NS | 61.7 ± 3.2 | 58 | Case series | NS | Tissue culture |
| Cui et al 2019[33] | 2019 | 2011.01−2017.07 | China | 40 | NS | 59.13 ± 2.22 | 55 | Case series | 15 | Tissue culture |
| Czuczman et al 2018[34] | 2018 | 1995.01−2016.12 | USA | 21 | 12−84 | 53.0 ± 19.7 | 57 | Case series | NS | Tissue culture |
| Devkota et al 2014[35] | 2014 | 2001.01−2009.12 | India | 42 | 16−75 | 51.6 (mean) | 79 | Case series | 23 | Tissue culture |
| Duan et al 2020[36] | 2020 | 2014.01−2016.12 | China | 45 | NS | 51.2  ±  14.6 | 60 | Case series | 45 | Tissue culture |
| Enoch et al 2008[37] | 2008 | 2001.07−2006.06 | UK | 27 | 1−81 | 66 (mean) | 67 | Case series | 4 | Tissue culture |
| Flamme et al 2000[38] | 2000 | 1988−1996 | Germany | 58 | 16−82 | 55 (mean) | 55 | Case series | 6 | Tissue culture |
| Vasant et al 2014[39] | 2014 | 2005.01−2013.10 | USA | 84 | NS | NS | NS | Case series | 42 | Tissue culture |
| Haaker et al 1997[40] | 1997 | 1990−1994 | Germany | 16 | 14−59 | 41.1 (mean) | 63 | Case series | NS | Tissue culture |
| Heary et al 1994[41] | 1994 | 1989.01−1992.07 | USA | 32 | 22−69 | NS | 78 | Case series | 20 | Tissue culture |
| Ho et al 2007[42] | 2007 | 1995−2002 | USA | 53 | 5.8−20.4 | 14.3 (mean) | NS | Case series | NS | Tissue culture |
| Hollern et al 2019[43] | 2019 | 2005.01−2015.03 | USA | 416 | 17−87 | 57.1 (mean) | 57 | Case series | NS | Tissue culture |
| Hosameldin et al 2022[44] | 2022 | 2019.03−2021.02 | Egypt | 40 | 15−75 | 49.8 ± 12.5 | 70 | Case series | NS | Tissue culture |
| Hu et al 2019[45] | 2019 | 2007.07−2017.12 | China | 15 | 31−85 | 63.2 ± 16.9 | 80 | Case series | 12 | Tissue culture |
| Huang et al 2022[46] | 2022 | 2016.11−2019.12 | China | 13 | 45−69 | 58.27 ± 7.83 | 38 | Case series | NS | Tissue culture |
| Jo et al 2016[47] | 2016 | 2006.01−2011.12 | USA | 30 | 39−80 | 57.1 ± 12.2 | 50 | Case series | 9 | Tissue culture |
| Kang et al 2015[48] | 2015 | 2005.01−2013.03 | South Korea | 344 | NS | NS | 59 | Case series | 192 | Tissue culture |
| Kang et al 2019[49] | 2019 | 2016.01−2017.06 | South Korea | 13 | 29−69 | 54.7 (mean) | 54 | Case series | NS | Tissue culture |
| Kehrer et al 2014[50] | 2014 | 1994.01−2009.05 | Denmark | 192 | 56.2−75.6 | 66.6 (mean) | 57 | Case series | 94 | Tissue culture |
| Kihira et al 2020[51] | 2020 | 2014.07−2019.08 | USA | 72 | NS | 63 ± 16 | 57 | Case series | 25 | Tissue culture |
| Kim et al 2015[52] | 2015 | 2005.01−2013.12 | South Korea | 128 | 53−76 | NS | 66 | Case series | 50 | Tissue culture |
| Kim et al 2011[53] | 2011 | 2004.01−2008.08 | South Korea | 31 | 38−78 | 58.19 (mean) | 71 | Case series | 1 | Tissue culture |
| Kobayashi et al 2018[54] | 2018 | 2012.01−2013.12 | Japan | 132 | NS | 45  ±  24 | 50 | Case-control | NS | Tissue culture |
| Kucuk et al 2017[55] | 2017 | 2008−2013 | Turkey | 20 | 32−74 | 56.3 (mean) | 55 | Case series | NS | Tissue culture |
| Kuo et al 2020[56] | 2020 | 2005−2019 | USA | 111 | 25−90 | NS | 50 | Case series | NS | Tissue culture |
| Lang et al 2021[57] | 2021 | 2000−2020 | Germany | 155 | NS | 66.1 ± 12.4 | 57 | Case-control | 25 | Tissue culture |
| Lazzeri et al 2010[58] | 2010 | 2005.04−2007.04 | Italy | 72 | 31−86 | 57.7 ± 14.3 | 40 | Case series | NS | Tissue culture |
| Li et al 2018[59] | 2018 | 2009.01−2011.12 | China | 102 | 18−87 | 61 (mean) | 64 | Case series | 34 | Tissue culture |
| Liao et al 2018[60] | 2018 | 2001.01−2015.12 | China | 18 | 63−90 | 73.4 (mean) | 11 | Case-control | NS | Tissue culture |
| Lin et al 2013[61] | 2013 | 2007.01−2009.12 | China | 17 | 30−67 | 49.2 ± 9.7 | 59 | Case series | NS | Tissue culture |
| Lin et al 2015[62] | 2015 | 2008.03−2010.06 | China | 22 | 43−71 | 54.7 ± 8.0 | 59 | Case series | NS | Tissue culture |
| Lu et al 2015[63] | 2015 | 2009.01−2011.06 | China | 28 | 37−86 | 60.4(mean) | 46 | Case series | NS | Tissue culture |
| Lucio et al 2000[64] | 2000 | 1995.09−1997.08 | USA | 20 | 33−84 | 50.4(mean) | 75 | Case series | NS | Tissue culture |
| Ma et al 2020[65] | 2020 | 1997.01−2017.01 | China | 13 | 13−68 | 40.85 ± 19.34 | 38 | Case series | 5 | Tissue culture |
| Marco et al 2009[66] | 2009 | NS | Spain | 40 | 1−88 | 58 (mean) | 60 | Case series | NS | Tissue culture |
| Marschall et al 2011[67] | 2011 | 2003.01−2007.07 | USA | 92 | NS | 57.7 ± 14.5 | 53 | Case series | 29 | Tissue culture |
| Masuda et al 2011[68] | 2011 | 1997−2006 | Japan | 30 | 17−78 | 63.1 ± 12.9 | 57 | Case-control | 3 | Tissue culture |
| McClelland et al 2016[69] | 2016 | 2008.09−2011.02 | USA | 40 | NS | NS | NS | RCT | NS | Tissue culture |
| McHenry et al 2002[70] | 2002 | 1950−1994 1972−1982 | USA | 253 | 10−85 | 60 (median) | 63 | Case series | 197 | Tissue culture |
| Menon et al 2014[71] | 2014 | 2010.07−2012.06 | Oman | 32 | 4−80 | 51 (mean) | 69 | Case series | 2 | Tissue culture |
| Nagata et al 1998[72] | 1998 | 1989.01−2004.12 | Japan | 23 | NS | NS | NS | Case series | NS | Tissue culture |
| Nakamura et al 2022[73] | 2022 | 2011.01−2019.03 | Japan | 14 | 64−79 | 72.7 ± 5.2 | 79 | Case series | NS | Tissue culture |
| Nam et al 2011[74] | 2011 | 2004.01−2009.12 | South Korea | 57 | 12−82 | 61.6 (mean) | 58 | Non-randomized | NS | Tissue culture |
| Onen et al 2015[75] | 2015 | 2008−2013 | Turkey | 19 | 32−75 | 54.6 (mean) | 53 | Case series | NS | Tissue culture |
| Park et al 2018[76] | 2018 | 2005.01−2016.11 | USA | 11 | 54−95 | 75.5 ± 11.2 | 45 | Case series | 2 | Tissue culture |
| Pazinato et al 2022[77] | 2022 | 2013.05−2021.02 | Brazil | 102 | 1−86 | 55.6(mean) | 59 | Case-control | NS | Tissue culture |
| Ravichandran et al 2023[78] | 2023 | 2017−2021 | India | 259 | 12−85 | 46 ± 15.5 | NS | Case series | NS | Tissue culture |
| Rieneck et al 1996[79] | 1996 | 1987−1995 | Denmark | 14 | 15−83 | 61.2 (mean) | 57 | Case series | NS | Tissue culture |
| Schömig et al 2022[80] | 2022 | 2006.01−2020.12 | Germany | 135 | NS | 65.9 ± 13.6 | 55 | Case series | 11 | Tissue culture |
| Sehn et al 2011[81] | 2011 | 2001.01−2007.03 | USA | 297 | 1−92 | 62 (mean) | 47 | Case series | NS | Tissue culture |
| Senker et al 2022[82] | 2022 | 2018.12−2020.01 | Austria | 392 | NS | 58.09 ± 14.83 | 52 | Case series | NS | Tissue culture |
| Shibayama et al 2010[83] | 2010 | 2006.09−2009.04 | Japan | 12 | 23−82 | 64.3 (mean) | 67 | Case series | NS | Tissue culture |
| Siam et al 2016[84] | 2016 | 1994−2012 | Germany | 23 | NS | 65.1 ± 10.9 | 43 | Case series | 2 | Tissue culture |
| Soultanis et al 2003[85] | 2003 | 1993−2000 | USA | 5 | 11−16 | 14 ± 0.9 | 40 | Case series | NS | Tissue culture |
| Stambough et al 1992[86] | 1992 | NS | USA | 75 | 18−74 | 60.13 ± 17.45 | 53 | Case series | NS | Tissue culture |
| Sullivan et al 2019[87] | 2019 | 2000−2015 | USA | 65 | NS | 15 ± 2.6 | NS | Case-control | NS | Tissue culture |
| Terreaux et al 2016[88] | 2016 | 2004.11−2014.06 | France | 63 | NS | 68.2 ± 13.0 | 54 | Case series | 11 | Tissue culture |
| Tong et al 2019[89] | 2019 | 2016.06−2017.07 | China | 11 | 40−70 | 60.7 (mean) | 91 | Case series | NS | Tissue culture |
| Tronnier et al 1992[90] | 1992 | 1986.09−1987.09  1988.01−1989.07 | Germany | 412 | NS | NS | NS | Case series | NS | Tissue culture |
| Viola et al 1997[91] | 1997 | 1980−1995 | USA | 8 | 12−65 | 26.5 ± 18.4 | NS | Case series | NS | Tissue culture |
| Viswanathan et al 2022[92] | 2022 | 2010−2019 | India | 13 | 28−71 | 57.3 ± 12.4 | 31 | Case series | NS | Tissue culture |
| Wang et al 2020[93] | 2020 | 2016.12−2017.11 | China | 14 | 42−74 | 49.1 (mean) | 57 | Case series | 3 | Tissue culture |
| Wang et al 2016[94] | 2016 | 2002.08−2012.08 | China | 41 | 28−88 | 55.2 (mean) | 71 | Non-randomized | 14 | Tissue culture |
| Wu et al 2021[95] | 2021 | 2014.12−2018.12 | China | 12 | 35−73 | 56.5 (mean) | 67 | Case series | NS | Tissue culture |
| Yang et al 2014[96] | 2014 | 2006.01−2010.12 | China | 21 | 39−87 | 56.5 (mean) | 67 | Case series | NS | Tissue culture |
| Yang et al 2008[97] | 2008 | 2001.01−2006.01 | China | 52 | 27−88 | 63 (mean) | 56 | Case series | NS | Tissue culture |
| Yoon et al 2010[98] | 2010 | 2003.05−2007.12 | South Korea | 45 | 30−81 | 58 (mean) | 53 | Case series | 20 | Tissue culture |
| Yoon et al 2015[99] | 2015 | 2011.01−2013.12 | South Korea | 177 | NS | 69(mean) | 50 | Case-control | 90 | Tissue culture |
| Yoshida et al 2022[100] | 2022 | 2019.10−2021.08 | Japan | 10 | 12−87 | 59.2 ± 24.4 | 82 | Case series | NS | Tissue culture |
| Yuan et al 2020[101] | 2020 | 2005.01−2013.12 | China | 10 | NS | 58.1 ± 12.3 | 30 | Case series | NS | Tissue culture |
| Zhang et al 2021[102] | 2021 | 2010.01−2020.07 | China | 19 | 29−90 | 73 (mean) | 26 | Case series | 10 | Tissue culture |
| Zheng et al 2023[103] | 2023 | 2012.01−2019.12 | China | 39 | NS | 63.0 ± 12.7 | 36 | Case series | 17 | Tissue culture |
| Zheng et al 2021[104] | 2021 | 2014.06−2017.07 | China | 21 | 46−75 | 58.1 ± 7.5 | 57 | Case series | 15 | Tissue culture |
| Beyer et al 2024[105] | 2024 | 2008−2020 | Germany | 133 | NS | 67 (mean) | 85 | Case series | NS | Tissue culture |
| Borde et al 2024[106] | 2024 | 2020.03−2023.06 | India | 25 | 6−77 | 45.92 ± 19.73 | 14 | Case series | NS | Tissue culture |
| Dai et al 2024[107] | 2024 | 2013.01−2020.01 | China | 453 | NS | 63.28 ± 14.95 | 120 | Case series | NS | Tissue culture |
| El Yaagoubi et al 2024[108] | 2024 | 2019.04−2022.11 | France | 36 | 30−73 | NS | 13 | Case series | NS | Tissue culture |
| Rico Nieto et al 2023[109] | 2023 | 2011−2018 | Spain | 1680 | NS | 59 (mean) | 19 | Case series | NS | Tissue culture |
| Li et al 2024[110] | 2024 | 2012−2020 | China | 12 | NS | 53 (mean) | 9 | Case series | NS | Tissue culture |
| Ikenaga et al 2023[111] | 2023 | 2014.01−2022.04 | Japan | 30 | NS | 62.76 ± 15.6 | 20 | Case series | NS | Tissue culture |
| Kang et al 2023[112] | 2023 | 2021.01−2021.11 | Korea | 132 | NS | 61.9 ± 15.8 | 62 | Case series | NS | Tissue culture |
| Kim et al 2023[113] | 2023 | 2010.01−2021.06 | Korea | 122 | NS | 67 ± 16.0 | 55 | Case series | NS | Tissue culture |
| Sampedro et al 2010[114] | 2010 | 2005.01−2007.04 | USA | 22 | 17−85 | 67 (mean) | 45 | Case-control | NS | Molecular biology |
| Sheikh et al 2017[115] | 2017 | 2014.01−2015.06 | Iran | 57 | 24−72 | 49 (mean) | 70 | Case-control | NS | Molecular biology |
| Xu et al 2022[116] | 2022 | 2020.03−2021.08 | China | 108 | 14−82 | 57.8 (mean) | 51 | Case-control | 37 | Molecular biology |
| Zhang et al 2022[117] | 2022 | 2021.11−2022.12 | China | 38 | 23−71 | 57.4 ± 12.9 | 76 | Case-control | NS | Molecular biology |
| Cheng et al 2023[118] | 2023 | 2021.01−2023.07 | China | 78 | NS | 54.80 ± 17.07 | 52 | Case series | NS | Molecular biology |
| Lin et al 2023[119] | 2023 | 2019.03−2021.10 | China | 39 | NS | 61.9 ± 15.8 | 19 | Case series | NS | Molecular biology |
| Afshari et al 2020[120] | 2020 | 2008−2018 | UK | 21 | 1−15 | 4.37 ± 1.07 | 43 | Case series | 3 | T+B# |
| Ahuja et al 2017[121] | 2017 | 2012.11−2014.10 | UK | 45 | 28−87 | NS | 58 | Case series | 6 | T+B |
| Bae et al 2017[122] | 2017 | 2005.01−2015.12 | South Korea | 141 | NS | 65.8 ± 11.7 | 60 | Case series | 87 | T+B |
| Bornemann et al 2015[123] | 2015 | 2004−2009 | Germany | 90 | 14−89 | 63.5 ± 17 | 59 | Case series | 22 | T+B |
| Cherasse et al 2003[124] | 2003 | 1991−2001 | France | 35 | NS | 54 ± 18.1 | 63 | Case series | NS | T+B |
| Cohen et al 2020[125] | 2020 | 2003−2017 | USA | 29 | 1−18 | 11 (mean) | 62 | Case series | 18 | T+B |
| Colmenero et al 2010[126] | 2010 | NS | Spain | 23 | 24−73 | 47.1 ± 15.8 | 65 | Case-control | NS | T+B |
| Cruza et al 2015[127] | 2015 | 2003.01−2013.10 | Spain | 51 | 22−85 | 66 (mean) | 73 | Case series | 35 | T+B |
| Dagirmanjian et al 1996[128] | 1996 | 1985.01−1993.06 | USA | 37 | NS | NS | NS | Case series | NS | T+B |
| Dayer et al 2018[129] | 2018 | 2004.01−2014.12 | Switzerland, Canada, France, Spain | 103 | NS | 40.8 ± 43.3 | 65 | Retrospective | 37 | T+B |
| Falakassa et al 2014[130] | 2014 | 2007.01−2012.12 | USA | 6 | 51−80 | 63 (mean) | 67 | Case series | 1 | T+B |
| Feki et al 2019[131] | 2019 | 1996.01−2015.12 | Tunisia | 67 | 18−86 | 55 ± 16 | 57 | Case series | 11 | T+B |
| Hasan et al 2021[132] | 2021 | 2016.09−2020.04 | Iraq | 40 | 32.6−40.1 | 36.4 ± 11.8 | 20 | Case series | NS | T+B |
| Kasalak et al 2018[133] | 2018 | 2008.07−2017.04 | Netherlands | 64 | 16−91 | 61.7 ± 16.5 | 53 | Case series | NS | T+B |
| Kaya et al 2021[134] | 2021 | 2012.01−2018.12 | Turkey | 343 | 18−89 | 43.56 ± 15.91 | 53 | Case series | 165 | T+B |
| Kono et al 2019[135] | 2019 | 2009.06−2015.06 | Japan | 24 | 46−88 | 69.2 ± 13.3 | 79 | Case series | NS | T+B |
| Kurt et al 2021[136] | 2021 | 2008−2019 | Turkey | 233 | 26−91 | 62.12 ± 14.3 | 48 | Case series | 54 | T+B |
| Lestin et al 2018[137] | 2018 | 2012.1−2013.12 | Germany | 57 | 44−92 | 70 (mean) | 72 | Case series | 13 | T+B |
| Navarro et al 2022[138] | 2022 | 2015.1−2019.11 | Spain | 39 | 50−83 | 66.9 (mean) | 67 | Case-control | 2 | T+B |
| Peckham et al 2021[139] | 2021 | 2010.11−2018.07 | USA | 96 | NS | 57.9 ± 14.5 | 71 | Case series | NS | T+B |
| Saravolatz et al 2018[140] | 2018 | 1969−2009 | USA | 173 | NS | 59.4 ± 11.9 | NS | Case series | NS | T+B |
| Sillevis et al 1999[141] | 1999 | 1993−1996 | Netherlands | 11 | 37−72 | 51.1 ± 3.10 | 45 | Case series | 8 | T+B |
| Stangenberg et al 2021[142] | 2021 | 2013−2018 | Germany | 211 | 19−89 | 64.6 ± 14.8 | 66 | Case series | NS | T+B |
| Turunc et al 2007[143] | 2007 | 2000.01−2005.01 | Turkey | 75 | 17−82 | 57.8 ± 14.8 | 53 | Case series | 54 | T+B |
| Weihe et al 2022[144] | 2022 | 2007.11−2017.07 | USA | 221 | NS | 60.18 ± 13.4 | 62 | Case series | NS | T+B |
| Winkler et al 2024[145] | 2024 | 2010.07−2021.10 | USA | 527 | NS | 60.7 ± 13.3 | 313 | Case series | 114 | T+B |
| Algarny et al 2023[146] | 2023 | 2021.07−2023.08 | Germany | 75 | 15−85 | 60.13 ± 17.45 | 36 | Case series | NS | T+B |
| Both et al 2023[147] | 2023 | 2018.02−2022.06 | Germany | 124 | NS | NS | NS | Case series | NS | T+M* |
| Li et al 2024[148] | 2024 | 2022.10−2023.12 | China | 27 | NS | 60.07 ± 12.61 | 13 | Case series | NS | T+M |
| Chen et al 2022[149] | 2022 | 2017.01−2019.02 | China | 43 | 43−84 | 63.1 ± 9.1 | 67 | Case series | 16 | T+B+M$ |
| Choi et al 2014[150] | 2014 | 2009.05−2010.12 2011.11−2012.08 | South Korea | 45 | 13−84 | 59 (mean) | 51 | Case series | 22 | T+B+M |
| Fuursted et al 2008[151] | 2008 | 2002.10−2003.10 | Denmark | 18 | 23−88 | 55 (mean) | 72 | Case series | NS | T+B+M |
| Huang et al 2023[152] | 2023 | 2018.08−2021.08 | China | 141 | NS | 54.18  ± 13.93 | 61 | Case series | NS | T+B+M |
| Kupila et al 2003[153] | 2003 | 1995−2000 | Turkey | 20 | ＞18 | NS | NS | Case series | NS | T+B+M |
| Lecouvet et al 2004[154] | 2004 | 2001.01−2003.06 | Belgium | 19 | 28−79 | 58 (mean) | 68 | Case series | NS | T+B+M |
| Ma et al 2021[155] | 2021 | 2020.06−2021.05 | China | 30 | 48−85 | 65.2  ±  9.0 | 53 | Case-control | NS | T+B+M |
| Wang et al 2023[156] | 2023 | 2020−2022 | China | 25 | 41−82 | 61.1 ± 2.6 | 56 | Case series | NS | T+B+M |

Note: #T+B: Tissue culture + Blood culture, *T+M: Tissue culture + Molecular biology, $T+B+M: Tissue culture + Blood culture + Molecular biology

**Table S4. JBI critical appraisal checklist**

|  | No. | Q1* | Q2 | Q3 | Q4 | Q5 | Q6 | Q7 | Q8 | score |
| --- | --- | --- | --- | --- | --- | --- | --- | --- | --- | --- |
| 1 | Tal et al 2020 | Y | Y | Y | Y | NS | NS | Y | U | 5 |
| 2 | Afshari et al 2020 | U | Y | Y | Y | NS | NS | Y | N | 4 |
| 3 | Ahuja et al 2017 | Y | Y | Y | Y | NS | NS | Y | Y | 6 |
| 4 | Albert et al 2013 | Y | N | Y | Y | NS | NS | Y | Y | 5 |
| 5 | Algarny et al 2023 | U | N | Y | Y | NS | NS | Y | Y | 4 |
| 6 | Ameri et al 2021 | N | Y | Y | Y | NS | NS | Y | N | 4 |
| 7 | Arya et al 1996 | N | Y | Y | Y | NS | NS | Y | N | 4 |
| 8 | Avenel et al 2021 | Y | N | Y | Y | NS | NS | Y | Y | 5 |
| 9 | Bae et al 2017 | Y | Y | Y | Y | NS | NS | Y | Y | 6 |
| 10 | Bornemann et al 2015 | N | Y | Y | Y | NS | NS | Y | U | 4 |
| 11 | Both et al 2023 | Y | N | Y | Y | NS | NS | Y | Y | 5 |
| 12 | Braun et al 2020 | Y | Y | Y | Y | NS | NS | Y | Y | 6 |
| 13 | Bürger et al 2019 | Y | N | Y | Y | NS | NS | Y | Y | 5 |
| 14 | Burkhard et al 2021 | Y | Y | Y | Y | NS | NS | Y | Y | 6 |
| 15 | Cannavale et al 2022 | N | Y | Y | Y | NS | NS | Y | Y | 5 |
| 16 | Callanan et al 2021 | Y | N | Y | U | NS | NS | Y | Y | 4 |
| 17 | Carlson et al 2020 | Y | N | Y | Y | NS | NS | Y | Y | 5 |
| 18 | Chang et al 2015 | Y | Y | Y | Y | NS | NS | Y | Y | 6 |
| 19 | Chen et al 2015 | N | Y | Y | Y | NS | NS | Y | N | 4 |
| 20 | Chen et al 2021 | Y | Y | Y | Y | NS | NS | Y | Y | 6 |
| 21 | Chen et al 2022 | Y | Y | Y | Y | NS | NS | Y | Y | 6 |
| 22 | Cherasse et al 2003 | N | N | Y | Y | NS | NS | Y | N | 3 |
| 23 | Choe et al 2014 | N | N | Y | Y | NS | NS | Y | Y | 4 |
| 24 | Choi et al 2010 | N | Y | Y | Y | NS | NS | Y | N | 4 |
| 25 | Choi et al 2014 | N | Y | Y | Y | NS | NS | Y | N | 4 |
| 26 | Cohen et al 2020 | Y | Y | Y | Y | NS | NS | Y | Y | 6 |
| 27 | Colmenero et al 2010 | N | N | Y | Y | NS | NS | Y | N | 3 |
| 28 | Cruza et al 2015 | Y | Y | Y | Y | NS | NS | Y | Y | 6 |
| 29 | Cui et al 2022 | Y | Y | Y | Y | NS | NS | Y | Y | 6 |
| 30 | Cui et al 2019 | Y | Y | Y | Y | NS | NS | Y | Y | 6 |
| 31 | Czuczman et al 2018 | Y | Y | Y | Y | NS | NS | Y | Y | 6 |
| 32 | Dagirmanjian et al 1996 | N | Y | Y | Y | N | N | Y | N | 4 |
| 33 | Devkota et al 2014 | Y | Y | Y | Y | NS | NS | Y | Y | 6 |
| 34 | Dayer et al 2018 | N | Y | Y | Y | NS | NS | Y | Y | 5 |
| 35 | Duan et al 2020 | Y | Y | Y | Y | NS | NS | Y | N | 5 |
| 36 | ENch et al 2008 | N | Y | Y | Y | NS | NS | Y | N | 4 |
| 37 | Falakassa et al 2014 | N | Y | Y | Y | NS | NS | Y | N | 4 |
| 38 | Feki et al 2019 | N | Y | Y | N | NS | NS | Y | Y | 4 |
| 39 | Flamme et al 2000 | N | N | U | N | NS | NS | Y | Y | 2 |
| 40 | Fuursted et al 2008 | N | Y | Y | Y | N | N | Y | Y | 5 |
| 41 | Vasant et al 2014 | N | Y | Y | Y | NS | NS | Y | N | 4 |
| 42 | Haaker et al 1997 | N | Y | Y | Y | NS | NS | Y | N | 4 |
| 43 | Hasan et al 2021 | Y | Y | Y | Y | NS | NS | Y | Y | 6 |
| 44 | Heary et al 1994 | N | N | Y | Y | NS | NS | Y | N | 3 |
| 45 | Ho et al 2007 | Y | Y | Y | Y | NS | NS | Y | Y | 6 |
| 46 | Hollern et al 2019 | N | Y | Y | Y | NS | NS | Y | Y | 5 |
| 47 | Hosameldin et al 2022 | N | Y | Y | Y | NS | NS | Y | Y | 5 |
| 48 | Hu et al 2019 | N | Y | Y | Y | NS | NS | Y | Y | 5 |
| 49 | Huang et al 2023 | Y | Y | Y | U | Y | N | Y | Y | 6 |
| 50 | Huang et al 2022 | Y | Y | Y | U | NS | NS | Y | Y | 5 |
| 51 | Jo et al 2016 | N | Y | Y | Y | Y | N | Y | U | 5 |
| 52 | Kang et al 2015 | Y | Y | Y | Y | NS | NS | Y | Y | 6 |
| 53 | Kang et al 2019 | N | Y | Y | U | NS | NS | Y | U | 3 |
| 54 | Kasalak et al 2018 | Y | Y | Y | U | Y | N | Y | Y | 6 |
| 55 | Kaya et al 2021 | N | Y | Y | Y | Y | N | Y | Y | 6 |
| 56 | Kehrer et al 2014 | Y | N | Y | Y | NS | NS | Y | Y | 5 |
| 57 | Kihira et al 2020 | Y | Y | Y | Y | NS | NS | Y | Y | 6 |
| 58 | Kim et al 2015 | Y | Y | Y | Y | Y | N | Y | Y | 7 |
| 59 | Kim et al 2011 | N | Y | Y | Y | NS | NS | Y | Y | 5 |
| 60 | Kobayashi et al 2018 | N | Y | Y | Y | Y | N | Y | Y | 6 |
| 61 | KoN et al 2019 | Y | Y | Y | U | NS | NS | Y | Y | 5 |
| 62 | Kucuk et al 2017 | N | Y | U | U | NS | NS | Y | Y | 3 |
| 63 | Kuo et al 2020 | Y | Y | Y | Y | NS | NS | Y | Y | 6 |
| 64 | Kupila et al 2003 | N | Y | Y | U | NS | NS | Y | U | 3 |
| 65 | Kurt et al 2021 | N | Y | Y | U | NS | NS | Y | Y | 4 |
| 66 | Lang et al 2021 | Y | Y | Y | Y | Y | N | Y | Y | 7 |
| 67 | Lazzeri et al 2010 | Y | Y | Y | Y | NS | NS | Y | Y | 6 |
| 68 | Lecouvet et al 2004 | Y | Y | Y | U | NS | NS | Y | U | 4 |
| 69 | Lestin et al 2018 | Y | Y | Y | N | NS | NS | Y | Y | 5 |
| 70 | Li et al 2018 | Y | Y | Y | Y | NS | NS | Y | Y | 6 |
| 71 | Liao et al 2018 | Y | Y | Y | U | NS | NS | Y | U | 4 |
| 72 | Lin et al 2013 | N | Y | Y | U | NS | NS | Y | Y | 4 |
| 73 | Lin et al 2015 | N | Y | Y | U | NS | NS | Y | Y | 4 |
| 74 | Lu et al 2015 | N | Y | Y | U | NS | NS | Y | U | 3 |
| 75 | Lucio et al 2000 | N | Y | Y | U | NS | NS | Y | U | 3 |
| 76 | Ma et al 2020 | N | Y | Y | U | NS | NS | Y | U | 3 |
| 77 | Ma et al 2021 | N | Y | Y | U | NS | NS | Y | Y | 4 |
| 78 | Marco et al 2009 | N | N | Y | U | NS | NS | Y | U | 2 |
| 79 | Marschall et al 2011 | Y | Y | Y | Y | Y | N | Y | Y | 7 |
| 80 | Masuda et al 2011 | N | Y | Y | U | NS | NS | Y | Y | 4 |
| 81 | McClelland et al 2016 | N | Y | Y | Y | Y | N | Y | U | 5 |
| 82 | McHenry et al 2002 | N | Y | Y | Y | NS | NS | Y | Y | 5 |
| 83 | MeNn et al 2014 | Y | Y | Y | U | NS | NS | Y | U | 4 |
| 84 | Nagata et al 1998 | N | Y | Y | U | NS | NS | Y | U | 3 |
| 85 | Nakamura et al 2022 | Y | Y | Y | Y | Y | N | Y | Y | 7 |
| 86 | Nam et al 2011 | N | Y | Y | U | Y | N | Y | Y | 5 |
| 87 | Navarro et al 2022 | N | Y | Y | U | NS | NS | Y | Y | 4 |
| 88 | Onen et al 2015 | N | Y | Y | U | NS | NS | Y | U | 3 |
| 89 | Park et al 2018 | N | Y | Y | U | NS | NS | Y | U | 3 |
| 90 | Pazinato et al 2022 | Y | Y | Y | U | NS | NS | Y | Y | 5 |
| 91 | Peckham et al 2021 | Y | Y | Y | U | NS | NS | Y | Y | 5 |
| 92 | Ravichandran et al 2023 | N | Y | Y | U | NS | NS | Y | Y | 4 |
| 93 | Rieneck et al 1996 | N | Y | Y | Y | NS | NS | Y | U | 4 |
| 94 | Saravolatz et al 2018 | N | Y | Y | Y | NS | NS | Y | U | 4 |
| 95 | Schömig et al 2022 | N | Y | Y | Y | Y | N | Y | Y | 6 |
| 96 | Sehn et al 2011 | Y | Y | Y | U | NS | NS | Y | Y | 5 |
| 97 | Senker et al 2022 | Y | N | Y | U | NS | NS | Y | Y | 4 |
| 98 | Shibayama et al 2010 | Y | N | U | U | NS | NS | Y | U | 2 |
| 99 | Siam et al 2016 | Y | Y | N | Y | NS | NS | Y | Y | 5 |
| 100 | Sillevis et al 1999 | N | N | N | Y | Y | N | Y | U | 3 |
| 101 | Soultanis et al 2003 | N | Y | Y | U | NS | NS | Y | U | 3 |
| 102 | Stambough et al 1992 | Y | N | Y | U | NS | NS | Y | U | 3 |
| 103 | Stangenberg et al 2021 | Y | Y | Y | Y | NS | NS | Y | Y | 6 |
| 104 | Sullivan et al 2019 | Y | N | Y | U | Y | N | Y | Y | 5 |
| 105 | Terreaux et al 2016 | Y | Y | Y | Y | Y | N | Y | Y | 7 |
| 106 | Tong et al 2019 | Y | Y | Y | Y | NS | N | Y | Y | 6 |
| 107 | Tronnier et al 1992 | N | N | Y | U | NS | N | Y | U | 2 |
| 108 | Turunc et al 2007 | Y | N | Y | Y | Y | Y | Y | Y | 7 |
| 109 | Viola et al 1997 | N | N | U | Y | NS | NS | Y | N | 2 |
| 110 | Viswanathan et al 2022 | Y | N | Y | U | N | N | Y | U | 3 |
| 111 | Wang et al 2020 | Y | N | Y | Y | N | N | Y | Y | 5 |
| 112 | Wang et al 2023 | Y | Y | Y | U | N | N | Y | Y | 5 |
| 113 | Wang et al 2016 | Y | Y | Y | Y | Y | N | Y | Y | 7 |
| 114 | Weihe et al 2022 | Y | N | Y | Y | Y | N | Y | Y | 6 |
| 115 | Wu et al 2021 | Y | Y | Y | Y | Y | N | Y | Y | 7 |
| 116 | Yang et al 2014 | Y | Y | Y | N | Y | N | Y | Y | 6 |
| 117 | Yang et al 2008 | Y | N | U | U | N | N | Y | U | 2 |
| 118 | Yoon et al 2010 | Y | Y | Y | Y | NS | N | Y | Y | 6 |
| 119 | Yoon et al 2015 | Y | Y | Y | Y | N | N | Y | Y | 6 |
| 120 | Yoshida et al 2022 | N | Y | Y | N | Y | N | Y | U | 4 |
| 121 | Yuan et al 2020 | Y | Y | Y | Y | Y | N | Y | U | 6 |
| 122 | Zhang et al 2021 | N | Y | Y | U | NS | NS | Y | Y | 4 |
| 123 | Zheng et al 2023 | N | N | Y | Y | NS | NS | Y | Y | 4 |
| 124 | Zheng et al 2021 | Y | Y | Y | Y | NS | NS | Y | Y | 6 |
| 125 | Ahl et al 1999 | N | Y | Y | N | NS | NS | Y | Y | 4 |
| 126 | Chandrasenan et al 2011 | N | Y | Y | U | NS | NS | Y | U | 3 |
| 127 | Chen et al 2020 | Y | N | Y | U | NS | NS | Y | N | 3 |
| 128 | Colip et al 2018 | Y | Y | Y | U | Y | N | Y | Y | 6 |
| 129 | Dholoo et al 2023 | Y | Y | Y | N | NS | NS | Y | Y | 5 |
| 130 | Dobran et al 2017 | N | Y | Y | Y | NS | NS | Y | Y | 5 |
| 131 | Guo et al 2021 | Y | Y | Y | U | NS | NS | Y | Y | 5 |
| 132 | Jean et al 2017 | Y | Y | Y | U | Y | N | Y | Y | 6 |
| 133 | Nakamura et al 2018 | Y | Y | Y | U | Y | Y | Y | Y | 7 |
| 134 | Rankine et al 2004 | N | Y | Y | U | NS | NS | Y | U | 3 |
| 135 | Rocha et al 2015 | N | Y | Y | U | NS | NS | Y | U | 3 |
| 136 | Tang et al 2022 | Y | N | Y | U | Y | N | Y | U | 4 |
| 137 | Wang et al 2012 | Y | Y | Y | U | Y | N | Y | U | 5 |
| 138 | Sampedro et al 2010 | N | Y | Y | Y | Y | N | Y | Y | 6 |
| 139 | Sheikh et al 2017 | Y | Y | Y | Y | NS | NS | Y | Y | 6 |
| 140 | Xu et al 2022 | Y | Y | Y | U | N | N | Y | Y | 5 |
| 141 | Zhang et al 2022 | N | Y | Y | Y | NS | NS | Y | Y | 5 |
| 142 | Zhang et al 2023 | N | Y | Y | U | NS | NS | Y | U | 3 |
| 143 | Winkler et al 2024 | Y | Y | Y | U | NS | NS | Y | Y | 5 |
| 144 | Beyer et al 2024 | N | Y | Y | N | Y | Y | Y | Y | 6 |
| 145 | Borde et al 2024 | Y | N | Y | Y | N | N | Y | N | 4 |
| 146 | Cheng et al 2023 | N | N | Y | Y | NS | NS | Y | Y | 4 |
| 147 | Dai et al 2024 | Y | N | Y | N | Y | Y | Y | Y | 6 |
| 148 | El Yaagoubi et al 2024 | N | N | Y | Y | N | N | Y | Y | 4 |
| 149 | Rico Nieto et al 2023 | N | Y | Y | Y | NS | NS | Y | Y | 5 |
| 150 | Ratiu et al 2024 | N | Y | Y | Y | N | N | Y | Y | 5 |
| 151 | Li et al 2024 | Y | Y | Y | Y | NS | NS | Y | Y | 6 |
| 152 | Li et al 2024 | Y | Y | Y | Y | NS | NS | Y | Y | 6 |
| 153 | Ikenaga et al 2023 | Y | Y | Y | Y | NS | NS | Y | Y | 6 |
| 154 | Kang et al 2023 | N | Y | Y | Y | NS | NS | Y | Y | 5 |
| 155 | Lin et al 2023 | Y | Y | Y | Y | NS | NS | Y | Y | 6 |
| 156 | Kim et al 2023 | N | Y | Y | Y | NS | NS | Y | N | 4 |

Abbreviation Y: Yes; N: No; U: Unclear; NS: Not Applicable.

Note: *Q1: Were the criteria for inclusion in the sample clearly defined? Y: The study provided explicit diagnostic criteria for defining a patient as having confirmed or suspected spinal infection (e.g., based on clinical symptoms, imaging findings, microbiological, or histological results).

Q2: Were the study subjects and the setting described in detail?

Q3: Was the exposure measured in a valid and reliable way?

Q4: Were objective, standard criteria used for measurement of the condition?

Q5: Were confounding factors identified?

Q6: Were strategies to deal with confounding factors stated?

Q7: Were the outcomes measured in a valid and reliable way?

Q8: Was appropriate statistical Analysis used?

**Table S5 Subgroup analyses of blood culture assays for the detection of *Staphylococcus aureus***

|  | **Studies (n)** | **Samples (n)** | **Detection Rate (%)** | **I²** | **P-val*** |
| --- | --- | --- | --- | --- | --- |
| Total |  |  |  |  |  |
| Study Quality |  |  |  |  |  |
| Low (0–3) | 8 | 127 | 12.9 (3.2–27.9) | 78.4 | 0.3260 |
| Moderate (4–6) | 37 | 3267 | 18.1 (12.8–24.0) | 93.4 | 0.4723 |
| High (7–8) | 1 | 126 | 31.7 (23.7–40.6) |  | Ref |
| TB Burden (per 100,000) |  |  |  |  |  |
| 31–100 | 7 | 386 | 15.2 (4.1–31.7) | 95.0 | 0.7438 |
| 0–30 | 39 | 3134 | 17.9 (12.8–23.7) | 91.6 | Ref |
| Publication Year |  |  |  |  |  |
| Before 2010 | 10 | 245 | 25.1 (9.1–45.7) | 88.5 | 0.2924 |
| After 2010 | 36 | 3275 | 16.4 (11.8–21.6) | 93.3 | Ref |
| Brucellosis Burden |  |  |  |  |  |
| Moderate (1.0–10.0) | 10 | 909 | 9.8 (6.6–13.5) | 61.5 | 0.0788 |
| Low (<1.0) | 36 | 2611 | 20 .0 (14.0–26.8) | 93.3 | Ref |
| Sample Size |  |  |  |  |  |
| >50 | 21 | 2948 | 20.0 (13.8–27.0) | 95.8 | 0.3515 |
| ≤50 | 25 | 572 | 15.3 (8.5–23.8) | 79.5 | Ref |
| Male (%) |  |  |  |  |  |
| >50 | 29 | 2639 | 15.4 (10.1–21.5) | 93.6 | 0.5499 |
| NS | 5 | 386 | 29.5 (20.2–39.6) | 77.5 | 0.3451 |
| ≤50 | 12 | 495 | 19.7 (7.8–35.5) | 85.0 | Ref |
| Fever Rate (%) |  |  |  |  |  |
| >25 | 8 | 1558 | 17.5 (6.3–32.8) | 97.5 | 0.6008 |
| NS | 18 | 1352 | 21.6 (13.9–30.5) | 88.8 | 0.1812 |
| ≤25 | 20 | 610 | 13.9 (7.9–21.2) | 72.8 | Ref |

Abbreviation: TB: Tuberculosis, Ref: Reference. *P-value of Univariable Meta-regression.

**Table S6 Subgroup analyses of tissue-based assays for the detection of *Staphylococcus aureus***

|  | **Studies (n)** | **Samples (n)** | **Detection Rate (%)** | **I²** | **P-val*** |
| --- | --- | --- | --- | --- | --- |
| Total |  |  |  |  |  |
| Study Quality |  |  |  |  |  |
| Low (0–3) | 22 | 2740 | 20.7 (12.3–30.5) | 98.6 | 0.8555 |
| Moderate (4–6) | 108 | 9250 | 15.7 (12.7–19.0) | 94.2 | 0.4901 |
| High (7–8) | 12 | 568 | 19.6 (12.9–27.3) | 96.0 | Ref |
| TB Burden (per 100,000) |  |  |  |  |  |
| 31–100 | 47 | 4970 | 14.5 (10.8–18.6) | 95.1 | 0.2835 |
| 101–200 | 4 | 148 | 18.7 (12.8–25.3) | 0 | 0.6711 |
| 0–30 | 91 | 7440 | 17.8 (13.9–22.1) | 96.4 | Ref |
| Publication Year |  |  |  |  |  |
| Before 2010 | 112 | 10214 | 15.5 (12.6–18.6) | 94.3 | 0.1060 |
| After 2010 | 30 | 2344 | 21.3 (14.1–29.5) | 97.5 | Ref |
| Brucellosis Burden |  |  |  |  |  |
| Moderate (1.0–10.0) | 100 | 3260 | 15.8 (11.6–20.5) | 93.5 | 0.6729 |
| Low (<1.0) | 42 | 9298 | 17.2 (13.7–21.0) | 96.6 | Ref |
| Sample Size |  |  |  |  |  |
| >50 | 85 | 5921 | 16.3 (12.6–20.3) | 96.2 | 0.6772 |
| ≤50 | 57 | 6637 | 17.5 (13.4–22.0) | 96.0 | Ref |
| Male (%) |  |  |  |  |  |
| >50 | 32 | 3948 | 13.0 (08.2–18.7) | 93.4 | 0.2244 |
| NS | 94 | 7846 | 17.3 (14.0–20.9) | 95.6 | 0.1142 |
| ≤50 | 16 | 764 | 21.8 (11.3–34.5) | 91.8 | Ref |
| Fever Rate (%) |  |  |  |  |  |
| >25 | 41 | 3662 | 12.8 (08.1–18.3) | 97.6 | 0.3820 |
| NS | 83 | 5929 | 19.0 (15.3–23.0) | 92.5 | 0.0528 |
| ≤25 | 18 | 2967 | 16.7 (10.3–24.4) | 95.4 | Ref |

Abbreviation: TB: Tuberculosis, Ref: Reference. *P-value of Univariable Meta-regression.

**Table S7. Subgroup analyses of molecular assays for the detection of *Staphylococcus aureus***

|  | **Studies (n)** | **Samples (n)** | **Detection Rate (%)** | **I²** | **P-val*** |
| --- | --- | --- | --- | --- | --- |
| Total |  |  |  |  |  |
| Study Design |  |  |  |  |  |
| Case series | 11 | 637 | 12.6 (9.3−16.4) | 66.5 | 0.5480 |
| Case-control | 6 | 273 | 11.0 (6.3−16.7) | 44.9 | Ref |
| TB Burden (per 100,000) |  |  |  |  |  |
| 31–100 | 11 | 592 | 13.7 (9.7−18.2) | 51.2 | 0.1980 |
| 0–30 | 6 | 318 | 9.6 (6.6−13.1) | 32.1 | Ref |
| Publication Year |  |  |  |  |  |
| 2011–2024 | 13 | 831 | 12.7 (9.6−12.6) | 86.9 | 0.2979 |
| 2000–2010 | 4 | 79 | 8.3 (3.3−15.3) | 0.0 | Ref |
| Brucellosis Burden |  |  |  |  |  |
| Low (<1.0) | 5 | 286 | 11.6 (7.2−16.9) | 27.9 | 0.8457 |
| Moderate (1.0–10.0) | 11 | 567 | 12.5 (8.7−17) | 49.0 | 0.7597 |
| High (10.0–50.0) | 1 | 910 | 12.0 (9.3−15) | 34.9 | Ref |
| Sample Size |  |  |  |  |  |
| >50 | 5 | 566 | 9.3 (7.1−11.8) | 48.3 | 0.0081 |
| ≤50 | 12 | 344 | 14.8 (10.5−19.7) | 30.9 | Ref |
| Male (%) |  |  |  |  |  |
| >50 | 3 | 620 | 13.5 (9.7−17.9) | 47.5 | 0.4085 |
| NS | 2 | 202 | 9.3 (5.7−13.7) | 15.4 | 0.8309 |
| ≤50 | 12 | 88 | 99.0 (4.6−17) | 0 | Ref |
| Fever Rate (%) |  |  |  |  |  |
| NS | 14 | 714 | 10.8 (8.7−13.2) | 78.2 | - |
| >25 | 3 | 196 | 17.2 (5.5−33.6) | 86.5 | - |

Abbreviation: TB: Tuberculosis, Ref: Reference. *P-value of Univariable Meta-regression.

**Table S8 Subgroup analyses of blood culture assays for the detection of *Mycobacterium tuberculosis***

|  | **Studies (n)** | **Samples (n)** | **Detection Rate (%)** | **I²** | **P-val*** |
| --- | --- | --- | --- | --- | --- |
| Total |  |  |  |  |  |
| Study Quality |  |  |  |  |  |
| Low (0–3) | 8 | 127 | 0.0 (0.0–0.8) | 0 | 0.1559 |
| Moderate (4–6) | 37 | 3267 | 0.0 (0.0–0.0) | 0 | 0.0618 |
| High (7–8) | 1 | 126 | 0.8 (0.0–4.3) |  | Ref |
| TB Burden (per 100,000) |  |  |  |  |  |
| 31–100 | 7 | 386 | 0.0 (0.0–0.3) | 0 | 0.7714 |
| 0–30 | 39 | 3134 | 0.0 (0.0–0.1) | 0 | Ref |
| Publication Year |  |  |  |  |  |
| Before 2010 | 10 | 245 | 0.0 (0.0–0.4) | 0 | 0.8150 |
| After 2010 | 36 | 3275 | 0.0 (0.0–0.1) | 0 | Ref |
| Brucellosis Burden |  |  |  |  |  |
| Moderate (1.0–10.0) | 10 | 909 | 0.0 (0.0–0.1) | 0 | 0.6134 |
| Low (<1.0) | 36 | 2611 | 0.0 (0.0–0.1) | 0 | Ref |
| Sample Size |  |  |  |  |  |
| >50 | 21 | 2948 | 0.0 (0.0–0.1) | 0 | 0.7209 |
| ≤50 | 25 | 572 | 0.0 (0.0–0.2) | 0 | Ref |
| Male (%) |  |  |  |  |  |
| >50 | 29 | 2639 | 0.0 (0.0–0.1) | 0 | 0.7025 |
| NS | 5 | 386 | 0.0 (0.0–0.2) | 0 | 1.0000 |
| ≤50 | 12 | 495 | 0.0 (0.0–0.2) | 0 | Ref |
| Fever Rate (%) |  |  |  |  |  |
| >25 | 8 | 1558 | 0.0 (0.0–0.1) | 0 | 1.0000 |
| NS | 18 | 1352 | 0.0 (0.0–0.2) | 0 | 0.4531 |
| ≤25 | 20 | 610 | 0.0 (0.0–0.2) | 0 | Ref |

Abbreviation: TB: Tuberculosis, Ref: Reference. *P-value of Univariable Meta-regression.

**Table S9 Subgroup analyses of tissue-based assays for the detection of *Mycobacterium tuberculosis***

|  | **Studies (n)** | **Samples (n)** | **Detection Rate (%)** | **I²** | **P-val*** |
| --- | --- | --- | --- | --- | --- |
| Total |  |  |  |  |  |
| Study Quality |  |  |  |  |  |
| Low (0–3) | 22 | 2740 | 1.7 (0.1–5.5) | 94.3 | 0.1466 |
| Moderate (4–6) | 108 | 9250 | 0.9 (0.4–1.6) | 79.9 | 0.0294 |
| High (7–8) | 12 | 568 | 5.5 (0.5–5.4) | 89.6 | Ref |
| TB Burden (per 100,000) |  |  |  |  |  |
| 31–100 | 47 | 4970 | 1.1 (0.2–2.7) | 86.6 | 0.8750 |
| 101–200 | 4 | 148 | 3.7 (0.0–3.3) | 67.2 | 0.4475 |
| 0–30 | 91 | 7440 | 1.2 (0.5–2.2) | 86.0 | Ref |
| Publication Year |  |  |  |  |  |
| Before 2010 | 30 | 2344 | 2.3 (0.3–5.8) | 92.9 | 0.2190 |
| After 2010 | 112 | 10214 | 1.0 (0.5–1.8) | 81.6 | Ref |
| Brucellosis Burden |  |  |  |  |  |
| Moderate (1.0–10.0) | 100 | 9298 | 1.2 (0.5–2.1) | 85.9 | 0.8534 |
| Low (<1.0) | 42 | 3260 | 1.3 (0.2–3.4) | 86.9 | Ref |
| Sample Size |  |  |  |  |  |
| >50 | 57 | 6637 | 0.9 (0.3–1.8) | 79.5 | 0.4810 |
| ≤50 | 85 | 5921 | 1.4 (0.5–2.8) | 88.5 | Ref |
| Male (%) |  |  |  |  |  |
| >50 | 94 | 7846 | 1.9 (0.9–3.2) | 85.4 | 0.0444 |
| NS | 16 | 764 | 0.5 (0.0–2.9) | 92.7 | 0.8621 |
| ≤50 | 32 | 3948 | 0.3 (0.0–1.1) | 70.6 | Ref |
| Fever Rate (%) |  |  |  |  |  |
| >25 | 18 | 2967 | 0.9 (0.0–2.9) | 81.6 | 0.4439 |
| NS | 83 | 5929 | 1.0 (0.3–2.0) | 87.1 | 0.2982 |
| ≤25 | 41 | 3662 | 1.9 (0.6–4.0) | 84.4 | Ref |

Abbreviation: TB: Tuberculosis, Ref: Reference. *P-value of Univariable Meta-regression.

**Table S10. Subgroup analyses of molecular assays for the detection of *Mycobacterium tuberculosis***

|  | **Studies (n)** | **Samples (n)** | **Detection Rate (%)** | **I²** | **P-val*** |
| --- | --- | --- | --- | --- | --- |
| Total |  |  |  |  |  |
| Study Design |  |  |  |  |  |
| Case series | 11 | 637 | 7.8 (2.2−16.5) | 92.8 | 0.3977 |
| Case-control | 6 | 273 | 13.7 (6.1−23.7) | 69.6 | Ref |
| TB Burden (per 100,000) |  |  |  |  |  |
| 31–100 | 11 | 592 | 16.8 (11.3−23.0) | 70.3 | 0.0001 |
| 0–30 | 6 | 318 | 1.2 (0.0−6.7) | 84.6 | Ref |
| Publication Year |  |  |  |  |  |
| 2011–2024 | 13 | 831 | 14.0 (7.9−21.4) | 91.5 | 0.0021 |
| 2000–2010 | 4 | 79 | 0.3 (0.0−2.7) | 3.0 | Ref |
| Brucellosis Burden |  |  |  |  |  |
| Low (<1.0) | 5 | 286 | 0.4 (0.0−2.3) | 37.2 | 0.0146 |
| Moderate (1.0–10.0) | 11 | 567 | 16.4 (10.6−23.3) | 70.6 | 0.9345 |
| High (10.0–50.0) | 1 | 910 | 9.7 (4.6−16.3) | 90.5 | Ref |
| Sample Size |  |  |  |  |  |
| >50 | 5 | 566 | 12.1 (2.7−26.9) | 96.5 | 0.5982 |
| ≤50 | 12 | 344 | 8.6 (3.1−16.5) | 78.8 | Ref |
| Male (%) |  |  |  |  |  |
| >50 | 3 | 620 | 14.1 (8.4−21.0) | 76.4 | 0.3224 |
| NS | 2 | 202 | 0.0 (0.0−0.5) | 0 | 0.0804 |
| ≤50 | 12 | 88 | 6.9 (0.0−27.3) | 86.0 | Ref |
| Fever Rate (%) |  |  |  |  |  |
| NS | 14 | 714 | 9.6 (3.8−17.6) | 91.5 | - |
| >25 | 3 | 196 | 10.2 (2.1−23.2) | 83.4 | - |

Abbreviation: TB: Tuberculosis, Ref: Reference. *P-value of Univariable Meta-regression.

**Table S11 Subgroup analyses of blood culture assays for the detection of *Brucella***

|  | **Studies (n)** | **Samples (n)** | **Detection Rate (%)** | **I²** | **P-val*** |
| --- | --- | --- | --- | --- | --- |
| Total |  |  |  |  |  |
| Study Quality |  |  |  |  |  |
| Low (0–3) | 8 | 127 | 0.3 (0.0–2.3) | 0 | 0.6489 |
| Moderate (4–6) | 37 | 3267 | 0.2 (0.0–0.6) | 68.2 | 0.6805 |
| High (7–8) | 1 | 126 | 0.0 (0.0–2.9) |  | Ref |
| TB Burden (per 100,000) |  |  |  |  |  |
| 31–100 | 7 | 386 | 0.0 (0.0–0.4) | 0 | 0.6495 |
| 0–30 | 39 | 3134 | 0.2 (0.0–0.7) | 67.1 | Ref |
| Publication Year |  |  |  |  |  |
| Before 2010 | 10 | 245 | 1.5 (0.0–8.7) | 76.4 | - |
| After 2010 | 36 | 3275 | 0.0 (0.0–0.0) | 0 | Ref |
| Brucellosis Burden |  |  |  |  |  |
| Moderate (1.0–10.0) | 10 | 909 | 1.0 (0.0–4.4) | 85.9 | 0.0063 |
| Low (<1.0) | 36 | 2611 | 0.0 (0.0–0.1) | 0 | Ref |
| Sample Size |  |  |  |  |  |
| >50 | 21 | 2948 | 0.3 (0.0–1.1) | 81.8 | 0.4214 |
| ≤50 | 25 | 572 | 0.1 (0.0–0.4) | 0 | Ref |
| Male (%) |  |  |  |  |  |
| >50 | 29 | 2639 | 0.2 (0.0–0.9) | 74.2 | 0.4357 |
| NS | 5 | 386 | 0.1 (0.0–1.3) | 11.8 | 0.6910 |
| ≤50 | 12 | 495 | 0.1 (0.0–0.6) | 0 | Ref |
| Fever Rate (%) |  |  |  |  |  |
| >25 | 8 | 1558 | 0.1 (0.0–5.8) | 91.6 | 0.0588 |
| NS | 18 | 1352 | 0.0 (0.0–0.2) | 0 | 0.9919 |
| ≤25 | 20 | 610 | 0.0 (0.0–0.4) | 0 | Ref |

Abbreviation: TB: Tuberculosis, Ref: Reference. *P-value of Univariable Meta-regression.

**Table S12 Subgroup analyses of tissue-based assays for the detection of *Brucella***

|  | **Studies (n)** | **Samples (n)** | **Detection Rate (%)** | **I²** | **P-val*** |
| --- | --- | --- | --- | --- | --- |
| Total |  |  |  |  |  |
| Study Quality |  |  |  |  |  |
| Low (0–3) | 22 | 2740 | 0.0 (0.0–0.2) | 11.9 | 0.5484 |
| Moderate (4–6) | 108 | 9250 | 0.0 (0.0–0.1) | 2.8 | 0.5104 |
| High (7–8) | 12 | 568 | 0.0 (0.0–0.2) | 0 | Ref |
| TB Burden (per 100,000) |  |  |  |  |  |
| 31–100 | 47 | 4970 | 0.0 (0.0–0.2) | 0 | 0.9301 |
| 101–200 | 4 | 148 | 0.0 (0.0–0.6) | 0 | 0.6817 |
| 0–30 | 91 | 7440 | 0.0 (0.0–0.1) | 2.5 | Ref |
| Publication Year |  |  |  |  |  |
| Before 2010 | 30 | 2344 | 0.0 (0.0–0.1) | 0 | 0.5006 |
| After 2010 | 112 | 10214 | 0.0 (0.0–0.1) | 15.7 | Ref |
| Brucellosis Burden |  |  |  |  |  |
| Moderate (1.0–10.0) | 42 | 3260 | 0.1 (0.0–0.3) | 49.9 | 0.7308 |
| Low (<1.0) | 100 | 9298 | 0.0 (0.0–0.1) | 0 | Ref |
| Sample Size |  |  |  |  |  |
| >50 | 57 | 6637 | 0.0 (0.0–0.0) | 0 | 0.1922 |
| ≤50 | 85 | 5921 | 0.1 (0.0–0.2) | 31.7 | Ref |
| Male (%) |  |  |  |  |  |
| >50 | 94 | 7846 | 0.0 (0.0–0.1) | 0 | 0.2658 |
| NS | 16 | 764 | 0.0 (0.0–0.1) | 0 | 0.6960 |
| ≤50 | 32 | 3948 | 0.0 (0.0–0.0) | 39.4 | Ref |
| Fever Rate (%) |  |  |  |  |  |
| >25 | 18 | 2967 | 0.0 (0.0–0.0) | 0 | 0.6531 |
| NS | 83 | 5929 | 0.1 (0.0–0.2) | 24.9 | 0.1307 |
| ≤25 | 41 | 3662 | 0.0 (0.0–0.1) | 0 | Ref |

Abbreviation: TB: Tuberculosis, Ref: Reference. *P-value of Univariable Meta-regression.

**Table S13 Subgroup analyses of molecular assays for the detection of *Brucella***

|  | **Studies (n)** | **Samples (n)** | **Detection Rate (%)** | **I²** | **P-val*** |
| --- | --- | --- | --- | --- | --- |
| Total |  |  |  |  |  |
| Study Design |  |  |  |  |  |
| Case series | 11 | 637 | 1.1 (0.1–3.4) | 69.2 | 0.5056 |
| Case-control | 6 | 273 | 2.3 (0.0–8.1) | 81.5 | Ref |
| TB Burden (per 100,000) |  |  |  |  |  |
| 31–100 | 11 | 592 | 2.5 (0.4–6.1) | 77.7 | 0.1266 |
| 0–30 | 6 | 318 | 0.3 (0.0–1.2) | 0 | Ref |
| Publication Year |  |  |  |  |  |
| 2011–2024 | 13 | 831 | 1.9 (0.3–4.7) | 79.1 | 0.8797 |
| 2000–2010 | 4 | 79 | 0.3 (0.0–2.7) | 3 | Ref |
| Brucellosis Burden |  |  |  |  |  |
| Low (<1.0) | 5 | 286 | 0.4 (0.0–1.4) | 0 | 0.6892 |
| Moderate (1.0–10.0) | 11 | 567 | 2.6 (0.5–6.3) | 75.7 | 0.2368 |
| High (10.0–50.0) | 1 | 910 | 1.5 (0.3–3.6) | 74.6 | Ref |
| Sample Size |  |  |  |  |  |
| >50 | 5 | 566 | 2.8 (0.3–8.0) | 88.6 | 0.3044 |
| ≤50 | 12 | 344 | 0.9 (0.0–3.2) | 58.9 | Ref |
| Male (%) |  |  |  |  |  |
| >50 | 3 | 620 | 1.8 (0.3–4.6) | 75.3 | 0.9689 |
| NS | 2 | 202 | 0.4 (0.0–1.8) | 0 | 0.5475 |
| ≤50 | 12 | 88 | 1.7 (0.0–14.3) | 82.9 | Ref |
| Fever Rate (%) |  |  |  |  |  |
| NS | 14 | 714 | 9.6 (3.8–17.6) | 91.5 | - |
| >25 | 3 | 196 | 10.2 (2.1–23.2) | 83.4 | - |

Abbreviation: TB: Tuberculosis, Ref: Reference. *P-value of Univariable Meta-regression.

**Table S14 Subgroup analyses of blood culture assays for the detection of *Escherichia coli***

|  | **Studies (n)** | **Samples (n)** | **Detection Rate (%)** | **I²** | **P-val*** |
| --- | --- | --- | --- | --- | --- |
| Total |  |  |  |  |  |
| Study Quality |  |  |  |  |  |
| Low (0–3) | 8 | 127 | 1.2 (0.0–3.7) | 0 | 0.2279 |
| Moderate (4–6) | 37 | 3267 | 1.8 (0.9–3.0) | 67.4 | 0.2985 |
| High (7–8) | 1 | 126 | 5.6 (2.3–11.1) |  | Ref |
| TB Burden (per 100,000) |  |  |  |  |  |
| 31–100 | 7 | 386 | 2.5 (0.3–6.7) | 72.6 | 0.5264 |
| 0–30 | 39 | 3134 | 1.6 (0.8–2.7) | 60.6 | Ref |
| Publication Year |  |  |  |  |  |
| Before 2010 | 10 | 245 | 0.9 (0.0–2.9) | 12.0 | 0.3808 |
| After 2010 | 36 | 3275 | 2.0 (1.0–3.2) | 67.7 | Ref |
| Brucellosis Burden |  |  |  |  |  |
| Moderate (1.0–10.0) | 10 | 909 | 1.3 (0.2–3.3) | 61.1 | 0.4846 |
| Low (<1.0) | 36 | 2611 | 2.0 (1.0–3.3) | 63.8 | Ref |
| Sample Size |  |  |  |  |  |
| >50 | 21 | 2948 | 1.4 (0.5–2.8) | 78.2 | 0.2370 |
| ≤50 | 25 | 572 | 2.8 (1.5–4.5) | 9.4 | Ref |
| Male (%) |  |  |  |  |  |
| >50 | 29 | 2639 | 1.7 (0.7–3.0) | 68.6 | 0.5635 |
| NS | 5 | 386 | 1.4 (0.0–6.7) | 73.9 | 0.5464 |
| ≤50 | 12 | 495 | 2.4 (0.9–4.5) | 20.2 | Ref |
| Fever Rate (%) |  |  |  |  |  |
| >25 | 8 | 1558 | 0.1 (0.0–0.7) | 55.6 | 0.5131 |
| NS | 18 | 1352 | 0.1 (0.0–0.4) | 63.4 | 0.7365 |
| ≤25 | 20 | 610 | 0.0 (0.0–0.3) | 45.2 | Ref |

Abbreviation: TB: Tuberculosis, Ref: Reference. *P-value of Univariable Meta-regression.

**Table S15 Subgroup analyses of tissue-based assays for the detection of *Escherichia coli***

|  | **Studies (n)** | **Samples (n)** | **Detection Rate (%)** | **I²** | **P-val*** |
| --- | --- | --- | --- | --- | --- |
| Total |  |  |  |  |  |
| Study Quality |  |  |  |  |  |
| Low (0–3) | 22 | 2740 | 2.2 (1.0–4.0) | 84.3 | 0.3726 |
| Moderate (4–6) | 108 | 9250 | 3.0 (2.1–4.1) | 74.1 | 0.1331 |
| High (7–8) | 12 | 568 | 0.9 (0.1–2.6) | 40.9 | Ref |
| TB Burden (per 100,000) |  |  |  |  |  |
| 31–100 | 47 | 4970 | 2.4 (1.3–4.0) | 76.9 | 0.6561 |
| 101–200 | 4 | 148 | 3.4 (0.4–9.1) | 41.4 | 0.8285 |
| 0–30 | 91 | 7440 | 2.8 (1.9–3.9) | 76.4 | Ref |
| Publication Year |  |  |  |  |  |
| Before 2010 | 30 | 2344 | 3.3 (1.7–5.4) | 79.0 | 0.4218 |
| After 2010 | 112 | 10214 | 2.5 (1.8–3.4) | 74.5 | Ref |
| Brucellosis Burden |  |  |  |  |  |
| Moderate (1.0–10.0) | 42 | 3260 | 2.9 (1.7–4.5) | 78.2 | 0.7231 |
| Low (<1.0) | 100 | 9298 | 2.6 (1.8–3.6) | 75.3 | Ref |
| Sample Size |  |  |  |  |  |
| >50 | 57 | 6637 | 2.6 (1.7–3.8) | 70.3 | 0.9318 |
| ≤50 | 85 | 5921 | 2.7 (1.8–3.9) | 78.6 | Ref |
| Male (%) |  |  |  |  |  |
| >50 | 94 | 7846 | 2.4 (1.6–3.3) | 75.2 | 0.6601 |
| NS | 16 | 764 | 5.2 (2.5–8.7) | 65.3 | 0.1682 |
| ≤50 | 32 | 3948 | 2.8 (1.3–4.9) | 77.8 | Ref |
| Fever Rate (%) |  |  |  |  |  |
| >25 | 18 | 2967 | 3.4 (1.2–6.7) | 60.4 | 0.2086 |
| NS | 83 | 5929 | 3.2 (2.2–4.4) | 74.6 | 0.0593 |
| ≤25 | 41 | 3662 | 1.7 (0.8–2.9) | 77.3 | Ref |

Abbreviation: TB: Tuberculosis, Ref: Reference. *P-value of Univariable Meta-regression.

**Table S16 Subgroup analyses of molecular assays for the detection of *Escherichia coli***

|  | **Studies (n)** | **Samples (n)** | **Detection Rate (%)** | **I²** | **P-val*** |
| --- | --- | --- | --- | --- | --- |
| Total |  |  |  |  |  |
| Study Design |  |  |  |  |  |
| Case series | 11 | 637 | 2.5 (1.0–4.6) | 44.7 | 0.6330 |
| Case-control | 6 | 273 | 1.1 (0.0–4.6) | 84.7 | Ref |
| TB Burden (per 100,000) |  |  |  |  |  |
| 31–100 | 11 | 592 | 2.9 (0.9–6.0) | 67.6 | 0.0801 |
| 0–30 | 6 | 318 | 0.3 (0.0–2.5) | 64.0 | Ref |
| Publication Year |  |  |  |  |  |
| 2011–2024 | 13 | 831 | 2.6 (0.8–5.2) | 70.0 | 0.0410 |
| 2000–2010 | 4 | 79 | 0.0 (0.0–1.2) | 0 | Ref |
| Brucellosis Burden |  |  |  |  |  |
| Low (<1.0) | 5 | 286 | 1.4 (0.0–5.0) | 52.2 | 0.4307 |
| Moderate (1.0–10.0) | 11 | 567 | 2.3 (0.5–5.4) | 70.7 | 0.2452 |
| High (10.0–50.0) | 1 | 910 | 1.7 (0.5–3.8) | 68.5 | Ref |
| Sample Size |  |  |  |  |  |
| >50 | 5 | 566 | 4.2 (0.9–9.6) | 80.7 | 0.0523 |
| ≤50 | 12 | 344 | 0.8 (0.0–2.6) | 39.1 | Ref |
| Male (%) |  |  |  |  |  |
| >50 | 3 | 620 | 1.8 (0.3–4.5) | 72.3 | 0.7578 |
| NS | 2 | 202 | 1.7 (0.0–10.6) | 68.9 | 0.8222 |
| ≤50 | 12 | 88 | 1.0 (0.0–8.4) | 70.8 | Ref |
| Fever Rate (%) |  |  |  |  |  |
| NS | 14 | 714 | 1.0 (0.1–2.7) | 58.3 | - |
| >25 | 3 | 196 | 7.6 (2.8–14.4) | 56.2 | - |

Abbreviation: TB: Tuberculosis, Ref: Reference. *P-value of Univariable Meta-regression.

**Table S17 Subgroup analyses of blood culture assays for the detection of *Klebsiella pneumoniae***

|  | **Studies (n)** | **Samples (n)** | **Detection Rate (%)** | **I²** | **P-val*** |
| --- | --- | --- | --- | --- | --- |
| Total |  |  |  |  |  |
| Study Quality |  |  |  |  |  |
| Low (0–3) | 8 | 127 | 0.0 (0.0–0.8) | 0 | 0.0672 |
| Moderate (4–6) | 37 | 3267 | 0.1 (0.0–0.4) | 18.8 | 0.0860 |
| High (7–8) | 1 | 126 | 1.6 (0.2–5.6) |  | Ref |
| TB Burden (per 100,000) |  |  |  |  |  |
| 31–100 | 7 | 386 | 1.2 (0.1–3.2) | 38.7 | 0.0013 |
| 0–30 | 39 | 3134 | 0.0 (0.0–0.2) | 0 | Ref |
| Publication Year |  |  |  |  |  |
| Before 2010 | 10 | 245 | 0.0 (0.0–0.6) | 0 | 0.5566 |
| After 2010 | 36 | 3275 | 0.2 (0.0–0.5) | 20.3 | Ref |
| Brucellosis Burden |  |  |  |  |  |
| Moderate (1.0–10.0) | 10 | 909 | 0.5 (0.0–2.1) | 34.0 | 0.2654 |
| Low (<1.0) | 36 | 2611 | 0.1 (0.0–0.3) | 0 | Ref |
| Sample Size |  |  |  |  |  |
| >50 | 21 | 2948 | 0.1 (0.0–0.3) | 15.2 | 0.1101 |
| ≤50 | 25 | 572 | 0.5 (0.1–1.3) | 0 | Ref |
| Male (%) |  |  |  |  |  |
| >50 | 29 | 2639 | 0.2 (0.0–0.5) | 20.9 | 0.5754 |
| NS | 5 | 386 | 0.0 (0.0–0.4) | 0 | 0.2541 |
| ≤50 | 12 | 495 | 0.3 (0.0–1.8) | 0 | Ref |
| Fever Rate (%) |  |  |  |  |  |
| >25 | 8 | 1558 | 0.2 (0.0–1.6) | 58.6 | 0.8684 |
| NS | 18 | 1352 | 0.1 (0.0–0.4) | 4.1 | 0.5604- |
| ≤25 | 20 | 610 | 0.2 (0.0–0.8) | 0 | Ref |

Abbreviation: TB: Tuberculosis, Ref: Reference. *P-value of Univariable Meta-regression.

**Table S18 Subgroup analyses of the diagnostic performance of tissue-based assays for *Klebsiella pneumoniae***

|  | **Studies (n)** | **Samples (n)** | **Detection Rate (%)** | **I²** | **P-val*** |
| --- | --- | --- | --- | --- | --- |
| Total |  |  |  |  |  |
| Study Quality |  |  |  |  |  |
| Low (0–3) | 22 | 2740 | 0.3 (0.0–1.0) | 57.3 | 0.6645 |
| Moderate (4–6) | 108 | 9250 | 0.5 (0.2–0.8) | 52.6 | 0.4530 |
| High (7–8) | 12 | 568 | 0.1 (0.0–0.6) | 28.4 | Ref |
| TB Burden (per 100,000) |  |  |  |  |  |
| 31–100 | 47 | 4970 | 0.4 (0.1–0.9) | 57.9 | 0.6685 |
| 101–200 | 4 | 148 | 0.0 (0.0–0.6) | 0 | 0.2374 |
| 0–30 | 91 | 7440 | 0.5 (0.2–0.8) | 51.0 | Ref |
| Publication Year |  |  |  |  |  |
| Before 2010 | 30 | 2344 | 0.4 (0.1–1.1) | 49.4 | 0.9546 |
| After 2010 | 112 | 10214 | 0.4 (0.2–0.7) | 52.8 | Ref |
| Brucellosis Burden |  |  |  |  |  |
| Moderate (1.0–10.0) | 42 | 3260 | 0.5 (0.1–1.1) | 66.7 | 0.7247 |
| Low (<1.0) | 100 | 9298 | 0.4 (0.2–0.7) | 40.2 | Ref |
| Sample Size |  |  |  |  |  |
| >50 | 57 | 6637 | 0.4 (0.1–0.8) | 43.1 | 0.7337 |
| ≤50 | 85 | 5921 | 0.4 (0.2–0.9) | 56.4 | Ref |
| Male (%) |  |  |  |  |  |
| >50 | 94 | 7846 | 0.4 (0.2–0.8) | 52.5 | 0.2460 |
| NS | 16 | 764 | 1.0 (0.1–2.7) | 60.0 | 0.0732 |
| ≤50 | 32 | 3948 | 0.2 (0.0–0.5) | 29.5 | Ref |
| Fever Rate (%) |  |  |  |  |  |
| >25 | 18 | 2967 | 0.5 (0.0–1.8) | 41.9 | 0.7308 |
| NS | 83 | 5929 | 0.5 (0.2–0.8) | 47.1 | 0.6472 |
| ≤25 | 41 | 3662 | 0.3 (0.0–0.9) | 59.4 | Ref |

Abbreviation: TB: Tuberculosis, Ref: Reference. *P-value of Univariable Meta-regression .

**Table S19 Subgroup analyses of molecular assays for the detection of *Klebsiella pneumoniae***

|  | **Studies (n)** | **Samples (n)** | **Detection Rate (%)** | **I²** | **P-val*** |
| --- | --- | --- | --- | --- | --- |
| Total |  |  |  |  |  |
| Study Design |  |  |  |  |  |
| Case series | 11 | 637 | 1.2 (0.0–4.2) | 78.4 | 0.9452 |
| Case-control | 6 | 273 | 1.4 (0.0–4.8) | 63.2 | Ref |
| TB Burden (per 100,000) |  |  |  |  |  |
| 31–100 | 11 | 592 | 2.3 (0.4–5.8) | 75.8 | 0.0847 |
| 0–30 | 6 | 318 | 0.0 (0.0–0.5) | 0 | Ref |
| Publication Year |  |  |  |  |  |
| 2011–2024 | 13 | 831 | 1.6 (0.2–4.3) | 79 | 0.4570 |
| 2000–2010 | 4 | 79 | 0.3 (0.0–2.7) | 4.7 | Ref |
| Brucellosis Burden |  |  |  |  |  |
| Low (<1.0) | 5 | 286 | 0.4 (0.0–2.3) | 37.6 | 0.6473 |
| Moderate (1.0–10.0) | 11 | 567 | 2.0 (0.2–5.6) | 76.8 | 0.3325 |
| High (10.0–50.0) | 1 | 910 | 1.3 (0.2–5.6) | 73.8 | Ref |
| Sample Size |  |  |  |  |  |
| >50 | 5 | 566 | 0.6 (0.0–2.7) | 78.6 | 0.4153 |
| ≤50 | 12 | 344 | 1.8 (0.1–5.2) | 71.3 | Ref |
| Male (%) |  |  |  |  |  |
| >50 | 3 | 620 | 2.1 (0.3–5.3) | 75.5 | 0.4064 |
| NS | 2 | 202 | 0.0 (0.0–0.5) | 0 | 0.6340 |
| ≤50 | 12 | 88 | 0.4 (0.0–3.4) | 28.8 | Ref |
| Fever Rate (%) |  |  |  |  |  |
| NS | 14 | 714 | 0.4 (0.0–1.4) | 41.5 | - |
| >25 | 3 | 196 | 8.0 (0.6–22.4) | 84.8 | - |

Abbreviation: TB: Tuberculosis, Ref: Reference. *P-value of Univariable Meta-regression.

**Table S20 Subgroup analyses of molecular assays for the detection of *Pseudomonas aeruginosa***

|  | **Studies (n)** | **Samples (n)** | **Detection Rate (%)** | **I²** | **P-val*** |
| --- | --- | --- | --- | --- | --- |
| Total |  |  |  |  |  |
| Study Design |  |  |  |  |  |
| Case series | 11 | 637 | 1.3 (0.3–3.0) | 49.4 | 0.3123 |
| Case-control | 6 | 273 | 0.4 (0.0–1.9) | 31.7 | Ref |
| TB Burden (per 100,000) |  |  |  |  |  |
| 31–100 | 11 | 592 | 1.3 (0.0.3–3) | 46.4 | 0.2774 |
| 0–30 | 6 | 318 | 0.4 (0.0–1.4) | 32.3 | Ref |
| Publication Year |  |  |  |  |  |
| 2011–2024 | 13 | 831 | 1.0 (0.3–2.2) | 46.3 | 0.7819 |
| 2000–2010 | 4 | 79 | 0.7 (0.0–5.7) | 52.4 | Ref |
| Brucellosis Burden |  |  |  |  |  |
| Low (<1.0) | 5 | 286 | 0.5 (0.0–1.6) | 41.5 | 0.4715 |
| Moderate (1.0–10.0) | 11 | 567 | 1.4 (0.4–3.1) | 41.7 | 0.1885 |
| High (10.0–50.0) | 1 | 910 | 0.9 (0.3–2.1) | 44.4 | Ref |
| Sample Size |  |  |  |  |  |
| >50 | 5 | 566 | 1.1 (0.3–2.5) | 41.7 | 0.7691 |
| ≤50 | 12 | 344 | 0.8 (0.0–2.7) | 49.3 | Ref |
| Male (%) |  |  |  |  |  |
| >50 | 3 | 620 | 1.1 (0.2–2.7) | 48.4 | 0.8090 |
| NS | 2 | 202 | 0.4 (0.0–1.8) | 0 | 0.7239 |
| ≤50 | 12 | 88 | 0.8 (0.0–7.1) | 64.8 | Ref |
| Fever Rate (%) |  |  |  |  |  |
| NS | 14 | 714 | 0.9 (0.2–2.3) | 47.2 | - |
| >25 | 3 | 196 | 1.1 (0.0–4.3) | 52.1 | - |

Abbreviation: TB: Tuberculosis, Ref: Reference. *P-value of Univariable Meta-regression.

**Table S21 Subgroup analyses of blood culture assays for the detection of *Pseudomonas aeruginosa***

|  | **Studies (n)** | **Samples (n)** | **Detection Rate (%)** | **I²** | **P-val*** |
| --- | --- | --- | --- | --- | --- |
| Total |  |  |  |  |  |
| Study Quality |  |  |  |  |  |
| Low (0–3) | 8 | 127 | 0.3 (0.0–2.1) | 0 | 0.3796 |
| Moderate (4–6) | 37 | 3267 | 0.1 (0.0–0.2) | 0 | 0.5948 |
| High (7–8) | 1 | 126 | 0.0 (0.0–2.9) |  | Ref |
| TB Burden (per 100,000) |  |  |  |  |  |
| 31–100 | 7 | 386 | 0.1 (0.0–0.9) | 0 | 0.5554 |
| 0–30 | 39 | 3134 | 0.1 (0.0–0.2) | 0 | Ref |
| Publication Year |  |  |  |  |  |
| Before 2010 | 10 | 245 | 0.0 (0.0–0.6) | 0 | 0.7325 |
| After 2010 | 36 | 3275 | 0.1 (0.0–0.3) | 0 | Ref |
| Brucellosis Burden |  |  |  |  |  |
| Moderate (1.0–10.0) | 10 | 909 | 0.0 (0.0–0.1) | 0 | 0.0945 |
| Low (<1.0) | 36 | 2611 | 0.1 (0.0–0.4) | 0 | Ref |
| Sample Size |  |  |  |  |  |
| >50 | 21 | 2948 | 0.1 (0.0–0.3) | 0 | 0.9186 |
| ≤50 | 25 | 572 | 0.1 (0.0–0.4) | 0 | Ref |
| Male (%) |  |  |  |  |  |
| >50 | 29 | 2639 | 0.1 (0.0–0.3) | 0 | 0.2831 |
| NS | 5 | 386 | 0.5 (0.0–1.8) | 22.3 | 0.1048 |
| ≤50 | 12 | 495 | 0.0 (0.0–0.2) | 0 | Ref |
| Fever Rate (%) |  |  |  |  |  |
| >25 | 8 | 1558 | 0.1 (0.0–0.7) | 55.6 | 0.6734 |
| NS | 18 | 1352 | 0.1 (0.0–0.4) | 0 | 0.6025 |
| ≤25 | 20 | 610 | 0.0 (0.0–0.3) | 0 | Ref |

Abbreviation: TB: Tuberculosis, Ref: Reference. *P-value of Univariable Meta-regression.

**Table S22 Subgroup analyses of tissue-based assays for the detection of *Pseudomonas aeruginosa***

|  | **Studies (n)** | **Samples (n)** | **Detection Rate (%)** | **I²** | **P-val&*** |
| --- | --- | --- | --- | --- | --- |
| Total |  |  |  |  |  |
| Study Quality |  |  |  |  |  |
| Low (0–3) | 22 | 2740 | 0.9 (0.1–2.5) | 79.5 | 0.9825 |
| Moderate (4–6) | 108 | 9250 | 0.4 (0.2–0.7) | 40.4 | 0.4703 |
| High (7–8) | 12 | 568 | 1.0 (0.0–3.3) | 54.5 | Ref |
| TB Burden (per 100,000) |  |  |  |  |  |
| 31–100 | 47 | 4970 | 0.4 (0.1–0.9) | 56.1 | 0.2551 |
| 101–200 | 4 | 148 | 2.0 (0.0–0.8) | 72.4 | 0.6193 |
| 0–30 | 91 | 7440 | 0.5 (0.2–0.9) | 53.1 | Ref |
| Publication Year |  |  |  |  |  |
| Before 2010 | 30 | 2344 | 0.4 (0.0–1.2) | 65.2 | 0.8392 |
| After 2010 | 112 | 10214 | 0.5 (0.3–0.8) | 51,0 | Ref |
| Brucellosis Burden |  |  |  |  |  |
| Moderate (1.0–10.0) | 42 | 3260 | 0.8 (0.3–1.6) | 59.4 | 0.2184 |
| Low (<1.0) | 100 | 9298 | 0.4 (0.2–0.7) | 50.6 | Ref |
| Sample Size |  |  |  |  |  |
| >50 | 57 | 6637 | 0.7 (0.3–1.2) | 51.1 | 0.3788 |
| ≤50 | 85 | 5921 | 0.4 (0.2–0.8) | 55.1 | Ref |
| Male (%) |  |  |  |  |  |
| >50 | 94 | 7846 | 0.5 (0.2–0.8) | 43.8 | 0.7984 |
| NS | 16 | 764 | 1.0 (0.1–3.1) | 60.9 | 0.4939 |
| ≤50 | 32 | 3948 | 0.5 (0.1–1.5) | 68.7 | Ref |
| Fever Rate (%) |  |  |  |  |  |
| >25 | 18 | 2967 | 0.6 (0.1–1.7) | 31.3 | 0.7379 |
| NS | 83 | 5929 | 0.5 (0.2–0.9) | 56.3 | 0.7814 |
| ≤25 | 41 | 3662 | 0.4 (0.1–1.0) | 55.9 | Ref |

Abbreviation: TB: Tuberculosis, Ref: Reference.  *P-value of Univariable Meta-regression.

**References**

1. Ahl T, Hedström M, von Heijne A, Hammers StieRNAtedt S. Acute spinal epidural abscess without concurrent spondylodiscitis. Successful closed treatment in 10 cases. Acta Orthop Scand. 1999;70(2):199-202.

2. Chandrasenan J, Klezl Z, Bommireddy R, Calthorpe D. Spondylodiscitis in children: a retrospective series. J Bone Joint Surg Br. 2011. 93(8): 1122-5.

3. Chen EL, Rosenberg M, Saran N, Ozgen B, Xie K, Mar WA. Paraspinal Fat Stranding as an Unexpected Finding on Body Computed Tomography: A Key to Early Detection of Spinal Osteomyelitis. J Clin Imaging Sci. 2020. 10: 6.

4. Colip CG, Lotfi M, Buch K, Holalkere N, Setty BN. Emergent spinal MRI in IVDU patients presenting with back pain: do we need an MRI in every case. Emerg Radiol. 2018. 25(3): 247-256.

5. Dholoo F, Sriramanarayanan A, Prasad S, et al. Spondylodiscitis-a cohort analysis of its identification and management. Int Orthop. 2023. 47(3): 813-818.

6. Dobran M, Marini A, Gladi M, Nasi D, Colasanti R, Benigni R, et al. Deep spinal infection in instrumented spinal surgery: diagnostic factors and therapy. G Chir. 2017;38(3):124-9.

7. Guo W, Wang M, Chen G, Chen KH, Wan Y, Chen B, et al. Early surgery with antibiotic medication was effective and efficient in treating pyogenic spondylodiscitis. BMC Musculoskelet Disord. 2021;22(1):288.

8. Jean M, Irisson JO, Gras G, Bouchand F, Simo D, Duran C, et al. Diagnostic delay of pyogenic vertebral osteomyelitis and its associated factors. Scand J Rheumatol. 2017;46(1):64-8.

9. Nakamura T, Morimoto T, Katsube K, Yamamori Y, Mashino J, Kikuchi K. Clinical characteristics of pyogenic spondylitis and psoas abscess at a tertiary care hospital: a retrospective cohort study. J Orthop Surg Res. 2018;13(1):302.

10. Rankine JJ, Barron DA, Robinson P, Millner PA, Dickson RA. Therapeutic impact of percutaneous spinal biopsy in spinal infection. Postgrad Med J. 2004;80(948):607-9.

11. Rocha A, Castro R, Santos J. Endocarditis and spondylodiscitis associated with tunneled cuffed hemodialysis catheters: hospitalizations with poor outcomes. Int J Artif Organs. 2015;38(4):173-7.

12. Tang X, Li J, Wang C, Liu F, Guo J, Tan J, et al. Antibiotic-loaded calcium sulfate beads in spinal surgery for patients with spondylodiscitis: a clinical retrospective study. BMC Musculoskelet Disord. 2022;23(1):270.

13. Wang Z, Lenehan B, Itshayek E, Boyd M, Dvorak M, Fisher C, et al. Primary pyogenic infection of the spine in intravenous drug users: a prospective observational study. Spine (Phila Pa 1976). 2012;37(8):685-92.

14. Zhang LZ, Zhang Y, Zhang ZC, Wang JF, Wang F, Zhang SM, et al. [Clinical manifestation and treatment strategy of intervertebral space infection after radiofrequency ablation of lumbar nucleus pulposus]. Zhongguo Gu Shang. 2023;36(9):884-9.

15. Ratiu IA, Moisa CF, Țiburcă L, Hagi-Islai E, Ratiu A, Bako GC, et al. Antimicrobial Treatment Challenges in the Management of Infective Spondylodiscitis Associated with Hemodialysis: A Comprehensive Review of Literature and Case Series Analysis. Antibiotics (Basel). 2024;13(3):284.

16. Ackerman T, Singer-Jordan J, Shani A, Rahamimov N. Intra-discal drain insertion for culture and drainage of pyogenic spondylodiscitis: A one-step diagnostic and therapeutic procedure. Interv Neuroradiol. 2020;26(1):105-10.

17. Albert HB, Lambert P, Rollason J, Sorensen JS, Worthington T, Pedersen MB, et al. Does nuclear tissue infected with bacteria following disc herniations lead to Modic changes in the adjacent vertebrae. Eur Spine J. 2013;22(4):690-6.

18. Ameri B, Shufflebarger HL, Emerson B, Asghar J, George SG, Ramchandran S. Late spinal infections following posterior spinal fusion in pediatric deformities: treatment using single-stage titanium implant exchange. Spine Deform. 2021;9(3):751-5.

19. Arya S, Crow WN, Hadjipaviou AG, Nauta HJ, Borowski AM, Vierra LA, et al. Percutaneous transpedicular management of discitis. J Vasc Interv Radiol. 1996;7(6):921-7.

20. Avenel G, Guyader P, Fiaux E, Alcaix D, Zarnitsky C, Pouplin-Jardin S, et al. Microbiological diagnosis of suspected vertebral osteomyelitis with a focus on the yield of percutaneous needle biopsy: a 10-year cohort study. Eur J Clin Microbiol Infect Dis. 2021;40(2):297-302.

21. Braun A, Germann T, Wünnemann F, Weber MA, Schiltenwolf M, Akbar M, et al. Impact of MRI, CT, and Clinical Characteristics on Microbial Pathogen Detection Using CT-Guided Biopsy for Suspected Spondylodiscitis. J Clin Med. 2019;9(1):32.

22. Bürger J, Akgün D, Strube P, Putzier M, Pumberger M. Sonication of removed implants improves microbiological diagnosis of postoperative spinal infections. Eur Spine J. 2019;28(4):768-74.

23. Burkhard MD, Loretz R, Uçkay I, Bauer DE, Betz M, Farshad M. Occult infection in pseudarthrosis revision after spinal fusion. Spine J. 2021;21(3):370-6.

24. Cannavale A, Santoni M, Nardis P, Lucatelli P, Corona M, Cannavale G, et al. Role of CT and MR imaging in the assessment of suspected spondylodiscitis and planning of needle biopsy. Radiol Med. 2022;127(9):1023-31.

25. Callanan TC, AbjoRNAon C, DiCarlo E, Henry M, Sama AA, Girardi FP, et al. Prevalence of Occult Infections in Posterior Instrumented Spinal Fusion. Clin Spine Surg. 2021;34(1):25-31.

26. Carlson BC, Hines JT, Robinson WA, Sebastian AS, Greenwood-Quaintance KE, Patel R, et al. Implant Sonication versus Tissue Culture for the Diagnosis of Spinal Implant Infection. Spine (Phila Pa 1976). 2020;45(9):E525-525E532.

27. Chang CY, Simeone FJ, Nelson SB, Taneja AK, Huang AJ. Is Biopsying the Paravertebral Soft Tissue as Effective as Biopsying the Disk or Vertebral Endplate? 10-Year Retrospective Review of CT-Guided Biopsy of Diskitis-Osteomyelitis. AJR Am J Roentgenol. 2015;205(1):123-9.

28. Chen HC, Huang TL, Chen YJ, Tsou HK, Lin WC, Hung CH, et al. A Minimally Invasive Endoscopic Surgery for Infectious Spondylodiscitis of the Thoracic and Upper Lumbar Spine in Immunocompromised Patients. Biomed Res Int. 2015;2015:780451.

29. Chen J, Xuan T, Lu Y, Lin X, Lv Z, Chen M. Outcome of one-stage percutaneous endoscopic debridement and lavage combined with percutaneous pedicle screw fixation for lumbar pyogenic spondylodiscitis. J Orthop Surg (Hong Kong). 2021;29(3):23094990211065579.

30. Choe H, Aota Y, Kobayashi N, Nakamura Y, Wakayama Y, Inaba Y, et al. Rapid sensitive molecular diagnosis of pyogenic spinal infections using methicillin-resistant Staphylococcus-specific polymerase chain reaction and 16S ribosomal RNA gene-based universal polymerase chain reaction. Spine J. 2014;14(2):255-62.

31. Choi KB, Lee CD, Lee SH. Pyogenic spondylodiscitis after percutaneous endoscopic lumbar discectomy. J Korean Neurosurg Soc. 2010;48(5):455-60.

32. Cui Y, Mi C, Wang B, Zheng B, Sun L, Pan Y, et al. Manual Homogenization Improves the Sensitivity of Microbiological Culture for Patients with Pyogenic Spondylitis. Infect Drug Resist. 2022;15:6485-93.

33. Cui YP, Mi C, Wang B, Pan YX, Lin YF, Shi XD. [Analysis of influencing factors for pathogen culture result in patients with pyogenic spondylitis]. Beijing Da Xue Xue Bao Yi Xue Ban. 2019;51(6):1042-7.

34. Czuczman GJ, Marrero DE, Huang AJ, Mandell JC, Ghazikhanian V, Simeone FJ. Diagnostic yield of repeat CT-guided biopsy for suspected infectious spondylodiscitis. Skeletal Radiol. 2018;47(10):1403-10.

35. Devkota P, Krishnakumar R, Renjith Kumar J. Surgical management of pyogenic discitis of lumbar region. Asian Spine J. 2014;8(2):177-82.

36. Duan K, Qin Y, Ye J, Zhang W, Hu X, Zhou J, et al. Percutaneous endoscopic debridement with percutaneous pedicle screw fixation for lumbar pyogenic spondylodiscitis: a preliminary study. Int Orthop. 2020;44(3):495-502.

37. Enoch DA, Cargill JS, Laing R, Herbert S, Corrah TW, Brown NM. Value of CT-guided biopsy in the diagnosis of septic discitis. J Clin Pathol. 2008;61(6):750-3.

38. Flamme CH, Frischalowski T, Gossé E. Advantages and limitations of conservative treatment of spondylitis and spondylodiscitis. ZEITSCHRIFT FUR RHEUMATOLOGIE. 2000;59(4):233-9.

39. Garg V, Kosmas C, Young PC, Togaru UK, Robbin MR. Computed tomography-guided percutaneous biopsy for vertebral osteomyelitis: a department's experience. Neurosurg Focus. 2014;37(2):E10.

40. Haaker RG, Senkal M, Kielich T, Krämer J. Percutaneous lumbar discectomy in the treatment of lumbar discitis. Eur Spine J. 1997;6(2):98-101.

41. Heary RF, Hunt CD, Krieger AJ, Vaid C. HIV status does not affect microbiologic spectrum or neurologic outcome in spinal infections. Surg Neurol. 1994;42(5):417-23.

42. Ho C, Skaggs DL, Weiss JM, Tolo VT. Management of infection after instrumented posterior spine fusion in pediatric scoliosis. Spine (Phila Pa 1976). 2007;32(24):2739-44.

43. Hollern DA, Woods BI, Shah NV, Schroeder GD, Kepler CK, Kurd MF, et al. Risk Factors for Pseudarthrosis After Surgical Site Infection of the Spine. Int J Spine Surg. 2019;13(6):507-14.

44. Hosameldin A, Hussein M, Abdelhalim E, Shehab M, Osman A. Surgical management of spontaneous thoracic and lumbar spondylodiscitis by fixation and debridement. Surg Neurol Int. 2022;13:44.

45. Hu SY, Hsieh MS, Chang YT, Huang CC, Tsai CA, Tsai CL, et al. Clinical features, management, and outcome of iliopsoas abscess associated with cardiovascular disorders: a hospital-based observational case series study. BMC Musculoskelet Disord. 2019;20(1):474.

46. Huang Q, Gu Q, Song J, Yan F, Lin X. The effectiveness of percutaneous endoscopic lumbar discectomy combined with external lumbar drainage in the treatment of intervertebral infections. Front Surg. 2022;9:975681.

47. Jo JE, Miller AO, Cohn MR, Nemani VM, Schneider R, Lebl DR. Evaluating the Diagnostic Yield of Computed Tomography-Guided Aspirations in Suspected Post-operative Spine Infections. HSS J. 2016;12(2):119-24.

48. Kang SJ, Jang HC, Jung SI, Choe PG, Park WB, Kim CJ, et al. Clinical characteristics and risk factors of pyogenic spondylitis caused by gram-negative bacteria. PLoS One. 2015;10(5):e0127126.

49. Kang T, Park SY, Lee SH, Park JH, Suh SW. Spinal epidural abscess successfully treated with biportal endoscopic spinal surgery. Medicine (Baltimore). 2019;98(50):e18231.

50. Kehrer M, Pedersen C, Jensen TG, Lassen AT. Increasing incidence of pyogenic spondylodiscitis: a 14-year population-based study. J Infect. 2014;68(4):313-20.

51. Kihira S, Koo C, Mahmoudi K, Leong T, Mei X, Rigney B, et al. Combination of Imaging Features and Clinical Biomarkers Predicts Positive Pathology and Microbiology Findings Suggestive of Spondylodiscitis in Patients Undergoing Image-Guided Percutaneous Biopsy. AJNR Am J Neuroradiol. 2020;41(7):1316-22.

52. Kim CJ, Kang SJ, Choe PG, Park WB, Jang HC, Jung SI, et al. Which tissues are best for microbiological diagnosis in patients with pyogenic vertebral osteomyelitis undergoing needle biopsy. Clin Microbiol Infect. 2015;21(10):931-5.

53. Kim SH, Lee JK, Jang JW, Seo BR, Kim TS, Kim SH. Laminotomy with continuous irrigation in patients with pyogenic spondylitis in thoracic and lumbar spine. J Korean Neurosurg Soc. 2011;50(4):332-40.

54. Kobayashi K, Ando K, Ito K, Tsushima M, Morozumi M, Tanaka S, et al. Prediction of surgical site infection in spine surgery from tests of nasal MRSA colonization and drain tip culture. Eur J Orthop Surg Traumatol. 2018;28(6):1053-7.

55. Kucuk A, Karademir M, Tumturk A, Ulutabanca H, Ercal BD, Senol S, et al. Surgical Strategies for Spondylodiscitis due to Lumbar Disc Surgery. Turk Neurosurg. 2017;27(1):95-8.

56. Kuo AH, Cho CH, Huang RY, Kim CJ, Lee TC. Target-specific yield rate and clinical utility of percutaneous tissue sampling in spinal infection. Clin Imaging. 2020;68:257-62.

57. Lang S, Frömming A, Walter N, Freigang V, Neumann C, Loibl M, et al. Is There a Difference in Clinical Features, Microbiological Epidemiology and Effective Empiric Antimicrobial Therapy Comparing Healthcare-Associated and Community-Acquired Vertebral Osteomyelitis. Antibiotics (Basel). 2021;10(11):1410.

58. Lazzeri E, Erba P, Perri M, Doria R, Tascini C, Mariani G. Clinical impact of SPECT/CT with In-111 biotin on the management of patients with suspected spine infection. Clin Nucl Med. 2010;35(1):12-7.

59. Li YD, Wong CB, Tsai TT, Lai PL, Niu CC, Chen LH, et al. Appropriate duration of post-surgical intravenous antibiotic therapy for pyogenic spondylodiscitis. BMC Infect Dis. 2018;18(1):468.

60. Liao JC, Lai PL, Chen LH, Niu CC. Surgical outcomes of infectious spondylitis after vertebroplasty, and comparisons between pyogenic and tuberculosis. BMC Infect Dis. 2018;18(1):555.

61. Lin Y, Chen WJ, Zhu WT, Li F, Fang H, Chen AM, et al. Single-level lumbar pyogenic spondylodiscitis treated with minimally invasive anterior debridement and fusion combined with posterior fixation via Wiltse approach. J Huazhong Univ Sci Technolog Med Sci. 2013;33(5):707-12.

62. Lin Y, Li F, Chen W, Zeng H, Chen A, Xiong W. Single-level lumbar pyogenic spondylodiscitis treated with mini-open anterior debridement and fusion in combination with posterior percutaneous fixation via a modified anterior lumbar interbody fusion approach. J Neurosurg Spine. 2015;23(6):747-53.

63. Lu ML, Niu CC, Tsai TT, Fu TS, Chen LH, Chen WJ. Transforaminal lumbar interbody debridement and fusion for the treatment of infective spondylodiscitis in the lumbar spine. Eur Spine J. 2015;24(3):555-60.

64. Lucio E, Adesokan A, Hadjipavlou AG, Crow WN, Adegboyega PA. Pyogenic spondylodiskitis: a radiologic/pathologic and culture correlation study. Arch Pathol Lab Med. 2000;124(5):712-6.

65. Ma CC, Wang ZY, Lin GZ. [Diagnosis and treatment of primary intraspinal abscess]. Beijing Da Xue Xue Bao Yi Xue Ban. 2020;52(2):275-80.

66. de Lucas EM, González Mandly A, Gutiérrez A, Pellón R, Martín-Cuesta L, Izquierdo J, et al. CT-guided fine-needle aspiration in vertebral osteomyelitis: true usefulness of a common practice. Clin Rheumatol. 2009;28(3):315-20.

67. Marschall J, Bhavan KP, Olsen MA, Fraser VJ, Wright NM, Warren DK. The impact of prebiopsy antibiotics on pathogen recovery in hematogenous vertebral osteomyelitis. Clin Infect Dis. 2011;52(7):867-72.

68. Masuda T, Miyamoto K, Hosoe H, Shimizu K. Comparative study on the efficacy of two-staged (posterior followed by anterior) surgical treatment using spinal instrumentation on pyogenic and tuberculotic spondylitis. Arch Orthop Trauma Surg. 2011;131(6):765-72.

69. McClelland S 3rd, Takemoto RC, Lonner BS, Andres TM, Park JJ, Ricart-Hoffiz PA, et al. Analysis of Postoperative Thoracolumbar Spine Infections in a Prospective Randomized Controlled Trial Using the Centers for Disease Control Surgical Site Infection Criteria. Int J Spine Surg. 2016;10:14.

70. McHenry MC, Easley KA, Locker GA. Vertebral osteomyelitis: long-term outcome for 253 patients from 7 Cleveland-area hospitals. Clin Infect Dis. 2002;34(10):1342-50.

71. Menon VK, Kumar KM, Al Ghafri K. One-stage biopsy, debridement, reconstruction, and stabilization of pyogenic vertebral osteomyelitis. Global Spine J. 2014;4(2):93-100.

72. Nagata K, Ohashi T, Ariyoshi M, Sonoda K, Imoto H, Inoue A. Percutaneous suction aspiration and drainage for pyogenic spondylitis. Spine (Phila Pa 1976). 1998;23(14):1600-6.

73. Nakamura S, Nakai T, Hosozawa K, Tanaka Y, Kishimoto K, Sakata K, et al. Treatment strategy for surgical site infection post posterior lumbar interbody fusion: A retrospective study. J Orthop. 2022;31:40-4.

74. Nam KH, Song GS, Han IH, Choi BK, Cha SH. Diagnostic Value of Biopsy Techniques in Lumbar Spondylodiscitis: Percutaneous Needle Biopsy and Open Biopsy. Korean J Spine. 2011;8(4):267-71.

75. Onen MR, Yuvruk E, Karagoz G, Naderi S. Efficiency of Hyperbaric Oxygen Therapy in Iatrogenic Spinal Infections. Spine (Phila Pa 1976). 2015;40(22):1743-8.

76. Park JW, Park SM, Lee HJ, Lee CK, Chang BS, Kim H. Infection following percutaneous vertebral augmentation with polymethylmethacrylate. Arch Osteoporos. 2018;13(1):47.

77. Pazinato LV, Urakawa FS, Setuguti DT, da Motta-Leal-Filho JM, de Menezes MR. Diagnostic Yield of Computed Tomography-Guided Procedures for Spondylodiscitis. Cardiovasc Intervent Radiol. 2022;45(6):800-7.

78. Ravichandran R, Amritanand R, Moses V, Kandagaddala M, Krishnan V, David KS, et al. Computed Tomography-Guided Spinal Biopsy in Suspected Infective Spondylodiscitis: An Institutional Review of Its Utility. Indian J Radiol Imaging. 2023;33(3):289-94.

79. Rieneck K, Hansen SE, Karle A, Gutschik E. Microbiologically verified diagnosis of infectious spondylitis using CT-guided fine needle biopsy. APMIS. 1996;104(10):755-62.

80. Schömig F, Li Z, Becker L, Vu-Han TL, Pumberger M, Diekhoff T. Gas within the Intervertebral Disc Does Not Rule Out Spinal Infection-A Case Series of 135 Patients with Spontaneous Spondylodiscitis. Diagnostics (Basel). 2022;12(5).scientic

81. Sehn JK, Gilula LA. Percutaneous needle biopsy in diagnosis and identification of causative organisms in cases of suspected vertebral osteomyelitis. Eur J Radiol. 2012;81(5):940-6.

82. Senker W, Aspalter S, Radl C, Pichler J, Doppler S, Weis S, et al. Frequency and characteristics of bacterial and viral low-grade infections of the intervertebral discs: a prospective, observational study. J Orthop Traumatol. 2022;23(1):15.

83. Shibayama M, Nagahara M, Kawase G, Fujiwara K, Kawaguchi Y, Mizutani J. New needle biopsy technique for lumbar pyogenic spondylodiscitis. Spine (Phila Pa 1976). 2010;35(23):E1347-9.

84. Siam AE, El Saghir H, Boehm H. Adjacent segment infection after surgical treatment of spondylodiscitis. J Orthop Traumatol. 2016;17(1):41-51.

85. Soultanis K, Mantelos G, Pagiatakis A, Soucacos PN. Late infection in patients with scoliosis treated with spinal instrumentation. Clin Orthop Relat Res. 2003;(411):116-23.

86. Stambough JL, Beringer D. Postoperative wound infections complicating adult spine surgery. J Spinal Disord. 1992;5(3):277-85.

87. Sullivan BT, Abousamra O, Puvanesarajah V, Jain A, Hadad MJ, Milstone AM, et al. Deep Infections After Pediatric Spinal Arthrodesis: Differences Exist with Idiopathic, Neuromuscular, or Genetic and Syndromic Cause of Deformity. J Bone Joint Surg Am. 2019;101(24):2219-25.

88. Terreaux W, Geoffroy M, Ohl X, Job L, Cart P, Eschard JP, et al. Diagnostic contribution of a second percutaneous needle biopsy in patients with spontaneous diskitis and negative blood cultures and first biopsy. Joint Bone Spine. 2016;83(6):715-9.

89. Tong YJ, Liu JH, Fan SW, Zhao FD. One-stage Debridement via Oblique Lateral Interbody Fusion Corridor Combined with Posterior Pedicle Screw Fixation in Treating Spontaneous Lumbar Infectious Spondylodiscitis: A Case Series. Orthop Surg. 2019;11(6):1109-19.

90. Tronnier V, Schneider R, Kunz U, Albert F, Oldenkott P. Postoperative spondylodiscitis: results of a prospective study about the aetiology of spondylodiscitis after operation for lumbar disc herniation. Acta Neurochir (Wien). 1992;117(3-4):149-52.

91. Viola RW, King HA, Adler SM, Wilson CB. Delayed infection after elective spinal instrumentation and fusion. A retrospective analysis of eight cases. Spine (Phila Pa 1976). 1997;22(20):2444-50; discussion 2450-1.

92. Viswanathan VK, Shetty AP, Kanna RM, Rajasekaran S. Use of antibiotic-impregnated beads in the treatment of lumbosacral surgical site infection: A retrospective case series. J Clin Orthop Trauma. 2022;32:101984.

93. Wang B, Chen C, Hua W, Ke W, Lu S, Zhang Y, et al. Minimally Invasive Surgery Oblique Lumbar Interbody Debridement and Fusion for the Treatment of Lumbar Spondylodiscitis. Orthop Surg. 2020;12(4):1120-30.

94. Wang YC, Wong CB, Wang IC, Fu TS, Chen LH, Chen WJ. Exposure of Prebiopsy Antibiotics Influence Bacteriological Diagnosis and Clinical Outcomes in Patients With Infectious Spondylitis. Medicine (Baltimore). 2016;95(15):e3343.

95. Wu S, Lin B, Li X, Chen S, Zhang H, Wu Z, et al. Single-stage debridement via autogenous iliac bone graft through the OLIF corridor and lateral fixation in treating spontaneous single-level lumbar pyogenic spondylodiscitis. BMC Musculoskelet Disord. 2021;22(1):947.

96. Yang SC, Chen WJ, Chen HS, Kao YH, Yu SW, Tu YK. Extended indications of percutaneous endoscopic lavage and drainage for the treatment of lumbar infectious spondylitis. Eur Spine J. 2014;23(4):846-53.

97. Yang SC, Fu TS, Chen LH, Chen WJ, Tu YK. Identifying pathogens of spondylodiscitis: percutaneous endoscopy or CT-guided biopsy. Clin Orthop Relat Res. 2008;466(12):3086-92.

98. Yoon SH, Chung SK, Kim KJ, Kim HJ, Jin YJ, Kim HB. Pyogenic vertebral osteomyelitis: identification of microorganism and laboratory markers used to predict clinical outcome. Eur Spine J. 2010;19(4):575-82.

99. Yoon YK, Jo YM, Kwon HH, Yoon HJ, Lee EJ, Park SY, et al. Differential diagnosis between tuberculous spondylodiscitis and pyogenic spontaneous spondylodiscitis: a multicenter descriptive and comparative study. Spine J. 2015;15(8):1764-71.

100. Yoshida A, Akazawa T, Torii Y, Ueno J, Iinuma M, Niki H. Diagnosis of Spinal Infection with Alpha-defensin Lateral Flow Test: A Preliminary Report. Spine Surg Relat Res. 2022;6(5):443-7.

101. Yuan S, Ma F, Wang Y, Gong P. Minimally invasive spine surgery in the treatment of pyogenic spondylodiscitis: an initial retrospective series study. Wideochir Inne Tech Maloinwazyjne. 2019;14(2):333-9.

102. Zhang S, Wang S, Wang Q, Yang J, Xu S. Debridement and corpectomy via single posterior approach to treat pyogenic spondylitis after vertebral augmentation. BMC Musculoskelet Disord. 2021;22(1):591.

103. Zheng HL, Li B, Song SK, Chen PB, Zheng XF, Jiang LS, et al. Safety and Efficacy of Polyetheretherketone (PEEK) Cages and Cadaveric Allografts in Transforaminal Lumbar Interbody Fusion (TLIF) for Treating Lumbar Pyogenic Spondylodiscitis. Mediators Inflamm. 2023;2023:5171620.

104. Zheng Q, Ying X, Jin Y, Zhu B, Shen J, Wang Y, et al. Treatment of single-segment suppurative spondylitis with the transforaminal endoscopic focal cleaning and drainage. J Spinal Cord Med. 2021;44(2):267-75.

105. Beyer F, Wenk B, Jung N, Bredow J, Eysel P, Yagdiran A. An investigation into the quality of life improvements after vertebral osteomyelitis depending on the status of pathogen detection. Acta Orthop Traumatol Turc. 2024;58(2):130-4.

106. Borde MD, Menon VK, Kanade UP, Rajale SS, Mane AV, Varma H. Drug eluting bioactive glass ceramics for fusion in spondylodiscitis: a pilot study. Neurosurg Rev. 2024;47(1):80.

107. Dai G, Li S, Yin C, Sun Y, Hou J, Luan L, et al. Culture-negative versus culture-positive in pyogenic spondylitis and analysis of risk factors for relapse. Br J Neurosurg. 2024;38(2):527-31.

108. El Yaagoubi Y, Lioret E, Thomas C, Loret JE, Simonneau A, Michaud-Robert AV, et al. Value of (18) F-FDG PET/CT to Identify Occult Infection in Presumed Aseptic Pseudarthrosis after Spinal Fusion: Correlation with Intraoperative Cultures. World J Nucl Med. 2024;23(1):17-24.

109. Rico Nieto A, Loeches Yagüe B, Quiles Melero I, Talavera Buedo G, Pizones J, Fernández-Baillo Sacristana N. Descriptive study of spinal instrumentation-related infections in a tertiary hospital. Rev Esp Cir Ortop Traumatol. 2024;68(3):201-8.

110. Li J, Jiang D, Chang Z. Investigating the efficacy of vacuum sealing drainage versus traditional negative pressure drainage in treating deep incision infections following posterior cervical internal fixation-a retrospective cohort study. Eur J Med Res. 2024;29(1):125.

111. Ikenaga S, Yunaiyama D, Yasutomi M, Nakamura I, Okubo M, Saguchi T, et al. Usefulness of Percutaneous Drainage in Determining the Causative Microorganism in Patients with Spondylodiscitis: A Retrospective Cross-Sectional Study. J Belg Soc Radiol. 2023;107(1):51.

112. Kang MS, You KH, Hwang JY, Cho TG, Yoon JH, Lee CS, et al. In Vivo Comparison of Positive Microbial Culture by Wound Irrigation Methods: Biportal Endoscopic Versus Open Microscopic Transforaminal Lumbar Interbody Fusion. Spine (Phila Pa 1976). 2024;49(13):941-9.

113. Kim JY, Oh BH, Kim IS, Hong JT, Sung JH, Lee HJ. The safety and effectiveness of lumbar drainage for cerebrospinal fluid leakage after spinal surgery. Neurochirurgie. 2023;69(6):101501.

114. Sampedro MF, Huddleston PM, Piper KE, Karau MJ, Dekutoski MB, Yaszemski MJ, et al. A biofilm approach to detect bacteria on removed spinal implants. Spine (Phila Pa 1976). 2010;35(12):1218-24.

115. Sheikh AF, Khosravi AD, Goodarzi H, Nashibi R, Teimouri A, Motamedfar A, et al. Pathogen Identification in Suspected Cases of Pyogenic Spondylodiscitis. Front Cell Infect Microbiol. 2017;7:60.

116. Xu L, Zhou Z, Wang Y, Song C, Tan H. Improved accuracy of etiological diagnosis of spinal infection by metagenomic next-generation sequencing. Front Cell Infect Microbiol. 2022;12:929701.

117. Zhang Y, Chen J, Yi X, Chen Z, Yao T, Tang Z, et al. Evaluation of the metagenomic next-generation sequencing performance in pathogenic detection in patients with spinal infection. Front Cell Infect Microbiol. 2022;12:967584.

118. Cheng H, Wu H, Tan N, Liu Z, Wang N, Chen N, et al. Diagnostic Efficacy of Metagenomic Next-Generation Sequencing in Patients with Spinal Infections: A Retrospective Study. Infect Drug Resist. 2023;16:7613-20.

119. Lin W, Xie F, Li X, Yang R, Lu J, Ruan Z, et al. Diagnostic performance of metagenomic next-generation sequencing and conventional microbial culture for spinal infection: a retrospective comparative study. Eur Spine J. 2023;32(12):4238-45.

120. Afshari FT, Rodrigues D, Bhat M, Solanki GA, Walsh AR, Lo WB. Paediatric spondylodiscitis: a 10-year single institution experience in management and clinical outcomes. Childs Nerv Syst. 2020;36(5):1049-54.

121. Ahuja N, Sharma H. The effectiveness of computed tomography-guided biopsy for the diagnosis of spondylodiscitis: an analysis of variables affecting the outcome. Eur Rev Med Pharmacol Sci. 2017;21(9):2021-6.

122. Bae JY, Kim CJ, Kim UJ, Song KH, Kim ES, Kang SJ, et al. Concordance of results of blood and tissue cultures from patients with pyogenic spondylitis: a retrospective cohort study. Clin Microbiol Infect. 2018;24(3):279-82.

123. Bornemann R, Müller-Broich JD, Deml M, Sander K, Wirtz DC, Pflugmacher R. [Diagnosis and Treatment of Spondylodiscitis/Spondylitis in Clinical Practice]. Z Orthop Unfall. 2015;153(5):540-5.

124. Cherasse A, Martin D, Tavernier C, Maillefert JF. Are blood cultures performed after disco-vertebral biopsy useful in patients with pyogenic infective spondylitis. Rheumatology (Oxford). 2003;42(7):913.

125. Cohen LL, Shore BJ, Williams KA, Hedequist DJ, Hresko MT, Emans JB, et al. Diagnosing and treating native spinal and pelvic osteomyelitis in adolescents. Spine Deform. 2020;8(5):1001-8.

126. Colmenero JD, Morata P, Ruiz-Mesa JD, Bautista D, Bermúdez P, Bravo MJ, et al. Multiplex real-time polymerase chain reaction: a practical approach for rapid diagnosis of tuberculous and brucellar vertebral osteomyelitis. Spine (Phila Pa 1976). 2010;35(24):E1392-6.

127. Raya Cruz M, Vilchez Rueda HH, Marinescu CI, Sarasíbar Ezcurra H, Riera Jaume M, Payeras Cifre A. Infectious spondylitis in the Balearic Islands: An analysis of 51 cases. Rev Clin Esp (Barc). 2015;215(5):251-7.

128. Dagirmanjian A, Schils J, McHenry M, Modic MT. MR imaging of vertebral osteomyelitis revisited. AJR Am J Roentgenol. 1996;167(6):1539-43.

129. Dayer R, Alzahrani MM, Saran N, Ouellet JA, Journeau P, Tabard-Fougère A, et al. Spinal infections in children: a multicentre retrospective study. Bone Joint J. 2018;100-B(4):542-8.

130. Falakassa J, Hirsch BP, Norton RP, Mendez-Zfass M, Eismont FJ. Case reviews of infections of the spine in patients with a history of solid organ transplantation. Spine (Phila Pa 1976). 2014;39(19):E1154-8.

131. Feki A, Akrout R, Masmoudi K, Sellami I, Ezzeddine M, Mnejja MA, et al. Infectious spondylodiscitis: A twenty-year experience from a single tertiary referral center. Egyptian Rheumatologist. 2019;41(3):231-5.

132. Hasan GA, Raheem HQ, Qutub A, Wais YB, Katran MH, Shetty GM. Management of Pyogenic Spondylodiscitis Following Nonspinal Surgeries: A Tertiary Care Center Experience. Int J Spine Surg. 2021;15(3):591-9.

133. Kasalak Ö, Wouthuyzen-Bakker M, Adams H, Overbosch J, Dierckx R, Jutte PC, et al. CT-guided biopsy in suspected spondylodiscitis: microbiological yield, impact on antimicrobial treatment, and relationship with outcome. Skeletal Radiol. 2018;47(10):1383-91.

134. Kaya S, Kaya S, Kavak S, Comoglu S. A disease that is difficult to diagnose and treat: evaluation of 343 spondylodiscitis cases. J Int Med Res. 2021;49(11):3000605211060197.

135. Kono M, Koda M, Abe T, Miura K, Nagashima K, Fujii K, et al. Percutaneous endoscopic discectomy might be effective in selected cases of pyogenic spondylitis. J Orthop Surg (Hong Kong). 2019;27(3):2309499019885446.

136. Kurt EK, Kandemir B, Erayman I, Bitirgen M. EVALUATION OF PYOGENIC, BRUCELLA AND TUBERCULOUS SPONDYLODISCITIS CASES. J Int Med Res. 2021;37(4):1933-40.

137. Lestin-BeRNAtein F, Tietke M, Briedigkeit L, Heese O. Diagnostics and antibiotic therapy for spondylodiscitis. J Med Microbiol. 2018;67(6):757-68.

138. Navarro-Navarro R, Suárez-Cabañas AH, Fernández-Varela T, Lorenzo-Rivero JA, Montesdeoca-Ara A. Analysis of results of open and percutaneous disc biopsy in the diagnosis of spondylodiscitis. Rev Esp Cir Ortop Traumatol. 2022;66(3):189-99.

139. Peckham ME, Shah LM, Johnson SM, Ryals E, Noda G, Hutchins TA. Defining Disc Biopsy Timing in Relation to Blood Culture Results for Inpatients with Suspected Discitis-Osteomyelitis. J Vasc Interv Radiol. 2021;32(1):121-7.

140. Saravolatz LD 2nd, Labalo V, Fishbain J, Szpunar S, Johnson LB. Lack of effect of antibiotics on biopsy culture results in vertebral osteomyelitis. Diagn Microbiol Infect Dis. 2018;91(3):273-4.

141. Sillevis Smitt P, Tsafka A, van den Bent M, de Bruin H, Hendriks W, Vecht C, et al. Spinal epidural abscess complicating chronic epidural analgesia in 11 cancer patients: clinical findings and magnetic resonance imaging. J Neurol. 1999;246(9):815-20.

142. Stangenberg M, Mende KC, Mohme M, Krätzig T, Viezens L, Both A, et al. Influence of microbiological diagnosis on the clinical course of spondylodiscitis. Infection. 2021;49(5):1017-27.

143. Turunc T, Demiroglu YZ, Uncu H, Colakoglu S, Arslan H. A comparative analysis of tuberculous, brucellar and pyogenic spontaneous spondylodiscitis patients. J Infect. 2007;55(2):158-63.

144. Weihe R, Taghlabi K, Lowrance M, Reeves A, Jackson SR, Burton DC, et al. Culture Yield in the Diagnosis of Native Vertebral Osteomyelitis: A Single Tertiary Center Retrospective Case Series With Literature Review. Open Forum Infect Dis. 2022;9(3):ofac026.

145. Winkler WL, George IA, Gandra S, Baker JC, Tomasian A, Northrup B, et al. Diagnostic efficacy and clinical impact of image-guided core needle biopsy of suspected vertebral osteomyelitis. Int J Infect Dis. 2024;144:107027.

146. Algarny S, Perera A, Egenolf P, Weber M, Heck V, Walter S, et al. Postoperative Surgical Site Infections in Spine Surgery: Can the Duration of Surgery Predict the Pathogen Spectrum. In Vivo. 2023;37(4):1688-93.

147. Both A, Christner M, Berinson B, Dreimann M, Viezens L, Lütgehetmann M, et al. The added value of a commercial 16S/18S-PCR assay (UMD-SelectNA, Molzym) for microbiological diagnosis of spondylodiscitis: an observational study. Diagn Microbiol Infect Dis. 2023;106(1):115926.

148. Li C, Xiao NS, Ke BY, Li S, Lin Y. Application of Metagenomic Next-Generation Sequencing in Suspected Spinal Infectious Diseases. World Neurosurg. 2024;185:e542-542e548.

149. Chen ZH, Wang X, Zhang Y, Wu ST, Wu YH, Shi Q, et al. Percutaneous Transforaminal Endoscopic Debridement and Drainage with Accurate Pathogen Detection for Infectious Spondylitis of the Thoracolumbar and Lumbar Spine. World Neurosurg. 2022;164:e1179-1179e1189.

150. Choi SH, Sung H, Kim SH, Lee SO, Lee SH, Kim YS, et al. Usefulness of a direct 16S rRNA gene PCR assay of percutaneous biopsies or aspirates for etiological diagnosis of vertebral osteomyelitis. Diagn Microbiol Infect Dis. 2014;78(1):75-8.

151. Fuursted K, Arpi M, Lindblad BE, Pedersen LN. Broad-range PCR as a supplement to culture for detection of bacterial pathogens in patients with a clinically diagnosed spinal infection. Scand J Infect Dis. 2008;40(10):772-7.

152. Huang H, Shi J, Zheng M, Su S, Chen W, Ming J, et al. Pathogen detection in suspected spinal infection: metagenomic next-generation sequencing versus culture. Eur Spine J. 2023;32(12):4220-8.

153. Kupila L, Rantakokko-Jalava K, Jalava J, Nikkari S, Peltonen R, Meurman O, et al. Aetiological diagnosis of brain abscesses and spinal infections: application of broad range bacterial polymerase chain reaction analysis. J Neurol Neurosurg Psychiatry. 2003;74(6):728-33.

154. Lecouvet F, Irenge L, Vandercam B, Nzeusseu A, Hamels S, Gala JL. The etiologic diagnosis of infectious discitis is improved by amplification-based DNA analysis. Arthritis Rheum. 2004;50(9):2985-94.

155. Ma C, Wu H, Chen G, Liang C, Wu L, Xiao Y. The potential of metagenomic next-generation sequencing in diagnosis of spinal infection: a retrospective study. Eur Spine J. 2022;31(2):442-7.

156. Wang G, Long J, Zhuang Y, Leng X, Zhang Y, Liu L, et al. Application of metagenomic next-generation sequencing in the detection of pathogens in spinal infections. Spine J. 2023;23(6):859-67.
